# Supplementary material for: Atlas of multilineage stem cell differentiation reveals TMEM88 as a developmental regulator of blood pressure
Source: Nat Commun. 2025 Feb 4;16:1356. doi: 10.1038/s41467-025-56533-2 (PMC11794859; doi:10.1038/s41467-025-56533-2)
Supplement: Supplementary file 1 — Supplementary Information [file 41467_2025_56533_MOESM1_ESM.pdf]

SUPPLEMENTARY FIGURES, TABLES, AND DATA

Supplementary Figure 1: Quality control and barcoding metrics of scRNA-seq datasets.

Supplementary Figure 2: Characterisation of atlas dataset with broad cell type clusters.

Supplementary Figure 3: TRIAGE-Cluster analysis to identify specific cell types in the atlas dataset.

Supplementary Figure 4: Annotation of early TRIAGE cell type peaks with reference to spatial gene expression domains in murine embryogenesis.

Supplementary Figure 5: Evaluation of WNT signalling and candidate genes regulating differentiation.

Supplementary Figure 6: *TMEM88* as a regulator of mesendodermal development in hiPSCs and mouse model.

Supplementary Figure 7: Gating strategy used for flow cytometry analysis.

Supplementary Table 1: Quality control and barcoding metrics in iPSC pilot scRNA-seq experiment.

Supplementary Table 2: Quality control and barcoding metrics in atlas of differentiation dataset.

Supplementary Data 1: Barcoding plasmid DNA sequence.

Supplementary Data 2: All CellChat significant ligand-receptor interactions. P-values for interactions are derived using a permutation test.

Supplementary Data 3: CellphoneDB cell-cell interaction enrichment tables.

Supplementary Data 4: Enrichment of all regulons' activity in each cluster from pySCENIC analysis.

Supplementary Data 5: TRIAGE genes used for identifying each cell type peak in the atlas dataset.

Supplementary Data 6: Top 100 TRIAGE identity-defining genes for each TRIAGE-Cluster cell type peak and their TRIAGE-ParseR gene cluster assignments in the atlas dataset.

Supplementary Data 7: Specificity of cell identity-defining TRIAGE-ParseR genes across all TRIAGE-Cluster cell type peaks in the atlas dataset.

Supplementary Data 8: GO terms enriched for each TRIAGE-ParseR gene cluster in each TRIAGE-Cluster cell type peak in the atlas dataset. Enrichment p-values determined by a one-tailed hypergeometric test and adjusted using Benjamini-Hochberg FDR correction).

Supplementary Data 9: SMR analysis results for the top 100 most enriched genes as well as all differentially Wnt-related genes' association to systolic and diastolic blood pressure in cardiovascular tissue.

SUPPLEMENTARY FIGURES & LEGENDS

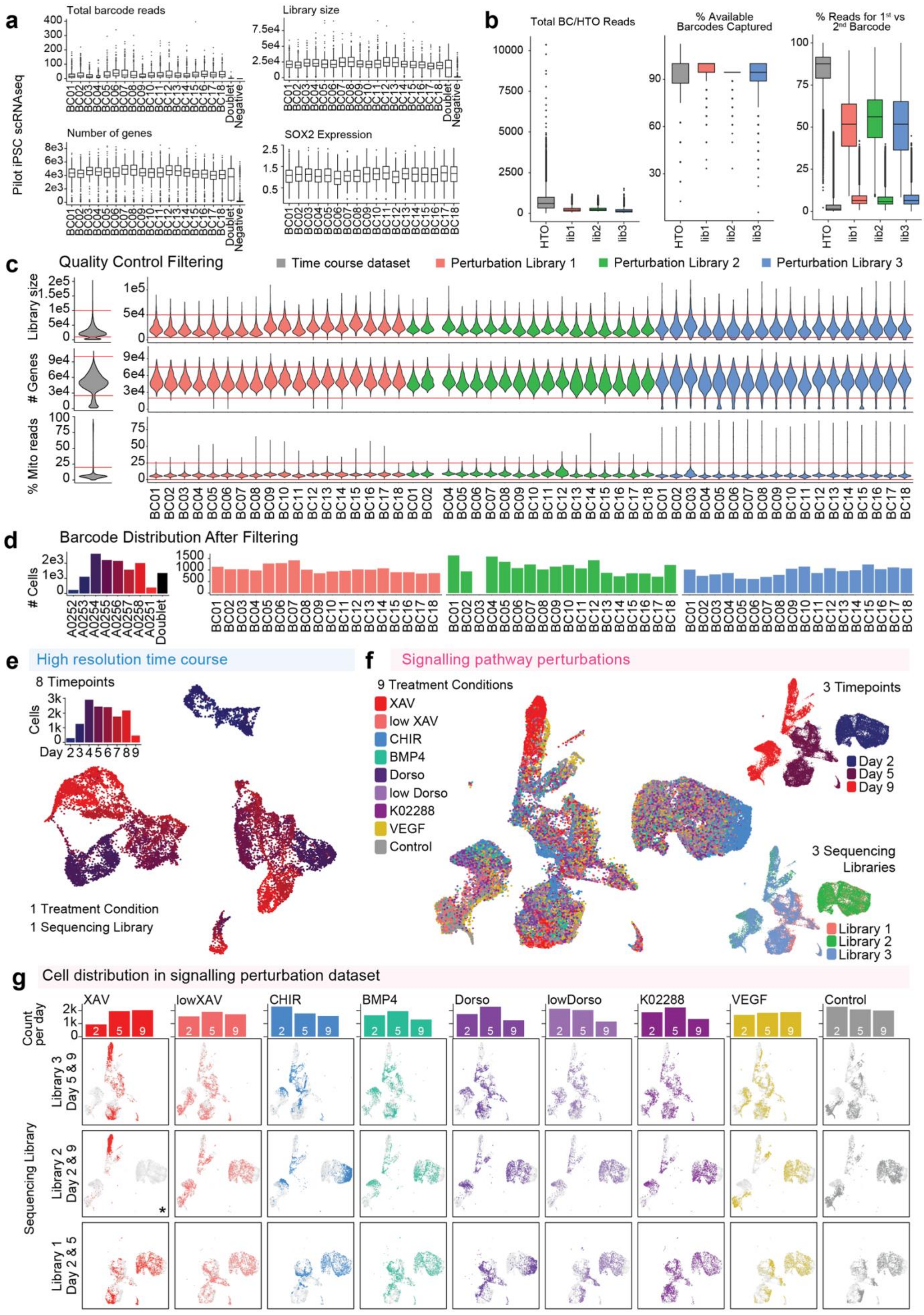

Supplementary Figure 1: Quality control of engineered barcoding cell lines. a) Box plots showing total number of genomic barcode reads (top left); total number of gene expression reads (library size, top right); total number of captured genes (bottom left); and expression of pluripotency gene *SOX2* in the pilot iPSC scRNA-seq experiment, grouped by the most highly mapped barcode per cell. See also **Supplementary Table 1**. **b)** Box plots showing total cell hashing (HTO) and genomic barcode (BC/libs 1-3) reads (left), percentage of total possible hashing or genomic barcodes captured (middle), and percentage of hashing or genomic barcode reads mapping to the two most highly mapped barcode per cell (right). See also **Supplementary Table 2**. **c)** Violin plots showing distribution of three quality control metrics and filtering thresholds chosen, compared between each cell line and scRNA-seq library. **d)** Distribution of cells after demultiplexing to allocate cells to each of the eight Cell Hashing antibodies, doublet, and negative cell barcode identities after filtering the cells for quality control metrics and reassignment of doublets not supported by multiple doublet detection algorithms (right). Bar plots showing number of cells annotated with an internal barcode versus doublet or negative classifications after re-allocation doublet cells not supported by multiple doublet detection algorithms. **e)** UMAP plot showing all high quality cells (13,682 cells total) in the time course data after filtering. Cells are coloured by time point. **f)** UMAP plot showing all high quality cells (48,526 cells total) in the signalling perturbation dataset after standard filtering and processing. Cells are coloured by treatment (upper), time point (lower left), and sequencing library (lower right). See **Table 1** for detailed breakdown of treatment, timepoint, and sequencing library combinations used. **g)** Distribution of cells from each treatment, timepoint, and sequencing library in the signalling perturbation dataset. * NB: one of the day 2 replicates in the XAV group was missing from the experiment (see also **Table 1**, **Supplementary Figure 1c & d**). Source data are provided as a Source data file.

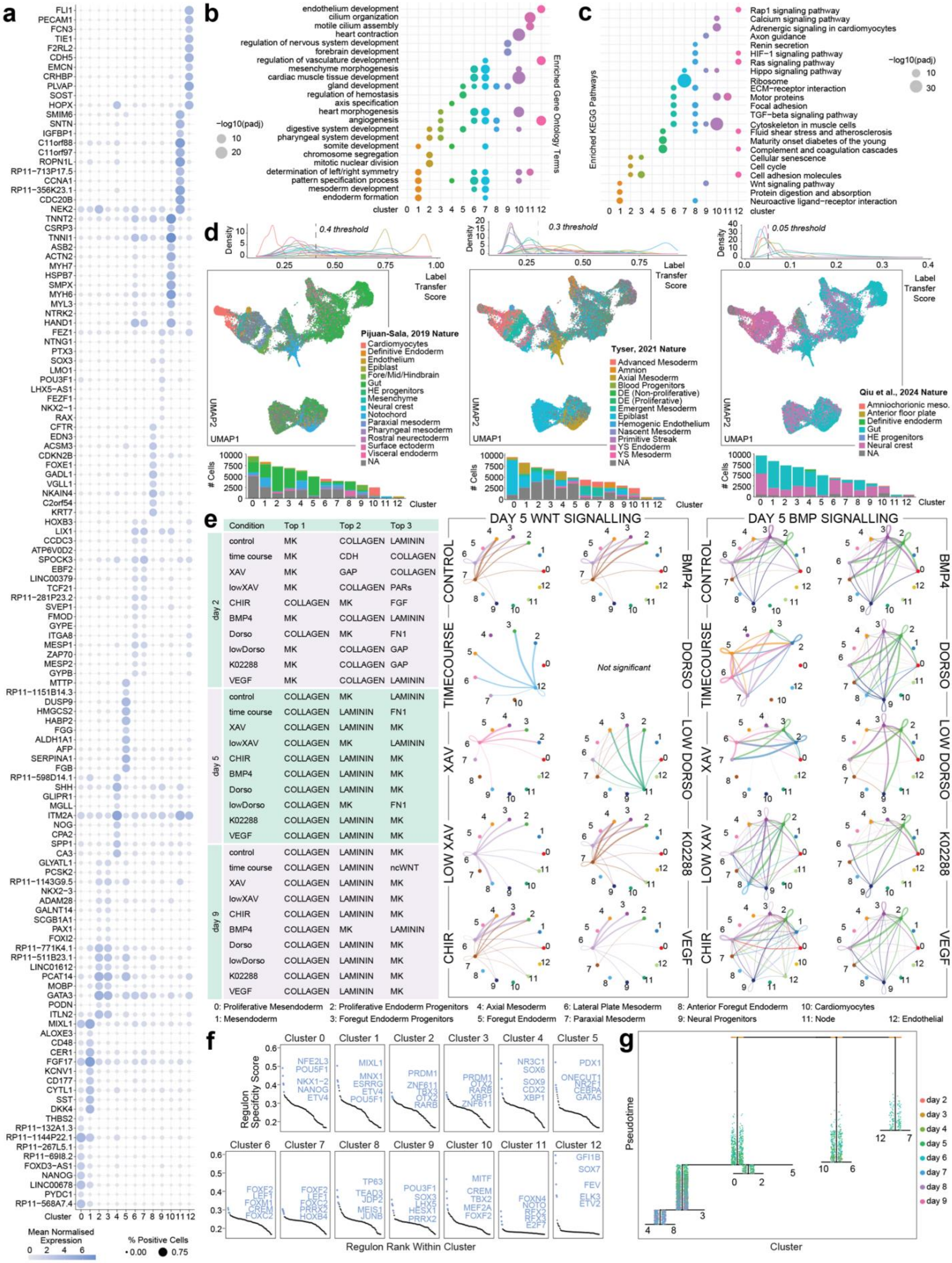

Supplementary Figure 2: Characterisation of atlas dataset with broad cell type clusters. a) Expression of marker genes for each cell cluster. Included genes were selected from known marker genes as well as the top 10 differentially

expressed genes for each cluster. **b-c)** Enrichment of select GO terms (**b**) and KEGG pathways (**c**) based on the top 100 most significantly differentially expressed genes for each cluster. Relevant terms and pathways were selected from the top 10 most significantly enriched results for each cluster. **d)** Label transfer results assessing similarity of each single-cell against *in vivo* cell types in mouse (left[1], right[2]) and human (middle[3]) embryogenesis. For each reference dataset we show distribution of label transfer prediction scores are shown with the selected threshold (top), a UMAP with cells with scores above the threshold coloured by their most confidence label (middle), and proportion of cells from each cluster annotated with each reference label (bottom). HE: Haematoendothelial; NA: Cells with scores below the threshold cut-off; DE: Definitive Endoderm; YS: Yolk Sac; Amniochorionic meso.: Amnichorionic mesoderm B. **e)** Results from CellChat predicting cell-cell interactions compared between treatment groups. The 3 pathways with the highest probabilities for each group at days 2, 5, and 9 of differentiation are displayed in the table (left), and significant predicted WNT- (centre) and BMP- (right) pathway communication between cell type clusters on day 5 are provided. See also **Supplementary Data 2** for full CellChat output of all predicted ligand-receptor interactions between clusters at all time points. **f)** Significantly enriched gene regulon activity in each cell cluster based on identification transcription factor-target co-expression modules and cis-regulatory motif enrichment analysis as part of the pySCENIC pipeline. Regulons are ranked by their specificity score (y-axis) and the top 5 most enriched regulons are highlighted. See also **Supplementary Data 4** for the whole enrichment output for all regulons in each cluster. **g)** Reconstruction of differentiation trajectories using the URD algorithm[4]. Clusters present at day 9 of differentiation were used as tips for tree construction. Source data are provided as a Source data file.

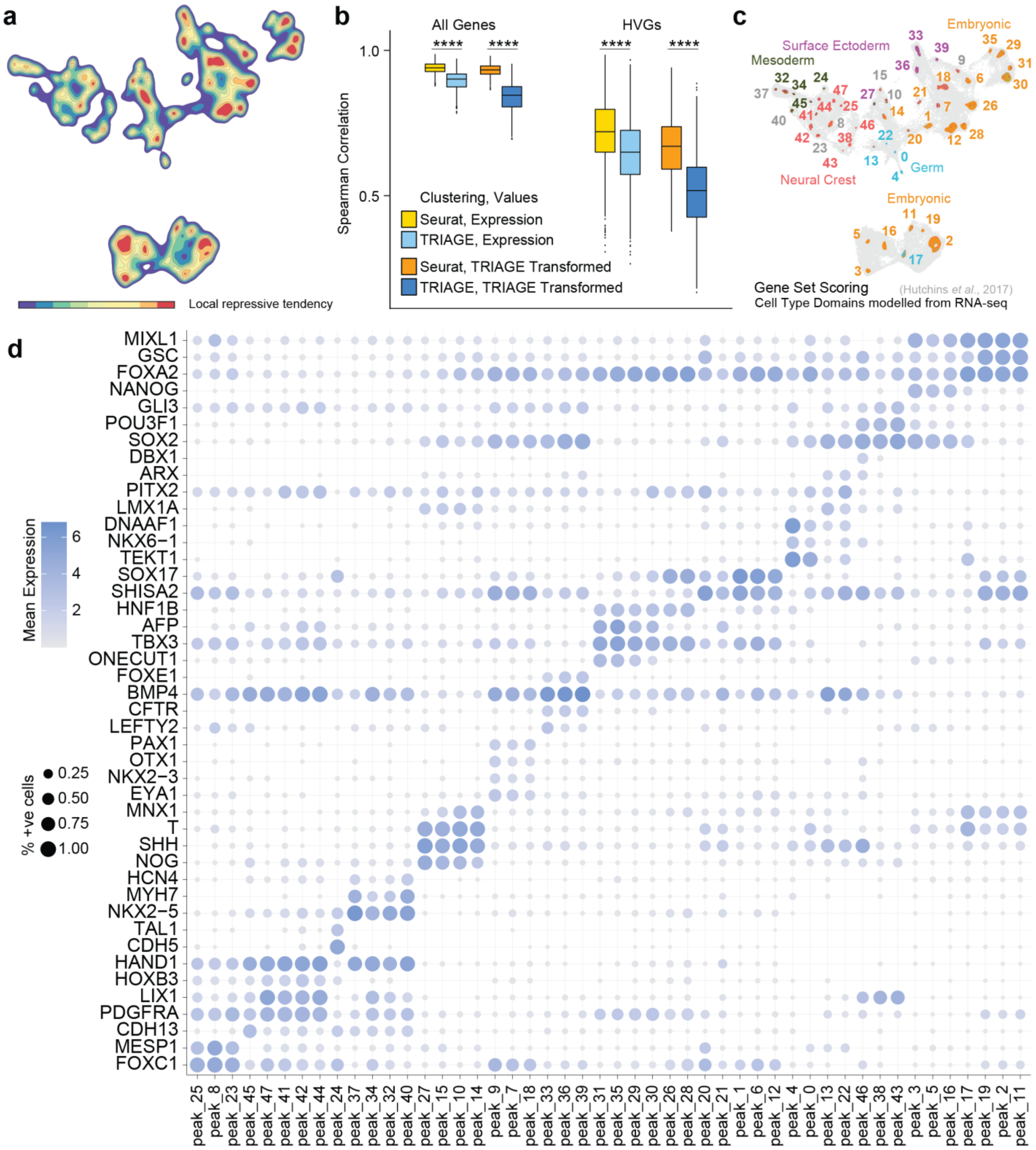

Supplementary Figure 3: TRIAGE-Cluster analysis to identify specific cell types in the atlas dataset. a) Contour plot showing regions of cells in the UMAP space expressing genes with high repressive tendency scores using bandwidth 0.25 to inform TRIAGE-Cluster[5] analysis. **b)** Comparison of peak-to-peak transcriptomic similarity (Spearman rank correlation) using all genes (left) and just the top 1000 highly variable genes (right), compared between Seurat clustering (resolution 2.1 to achieve the same number of clusters identified using TRIAGE-Cluster) and TRIAGE-Cluster, before and after TRIAGE transformation[6] of the expression values. ****p<2.22e-16 and n=1128 pairwise correlation values for all groups. **c)** Annotation of TRIAGE cell type peaks by reference to broad cell type domains from gene sets from 921 mouse tissue RNA-seq samples[7] using Seurat gene set scoring. Cells are coloured on a single-cell basis by their

assigned label after thresholding (threshold = 0). Dark grey cells do not have a label above threshold. Peak number labels are coloured by the label that makes up the greatest proportion of cells in that peak, where grey labels indicate peaks where most cells do not score above the threshold. Light grey cells do not fall within a peak. See also **Figure 3b-c**. **d)** Expression of select marker genes for each TRIAGE cell type peak to support annotations in **Figure 3h**. Source data are provided as a Source data file.

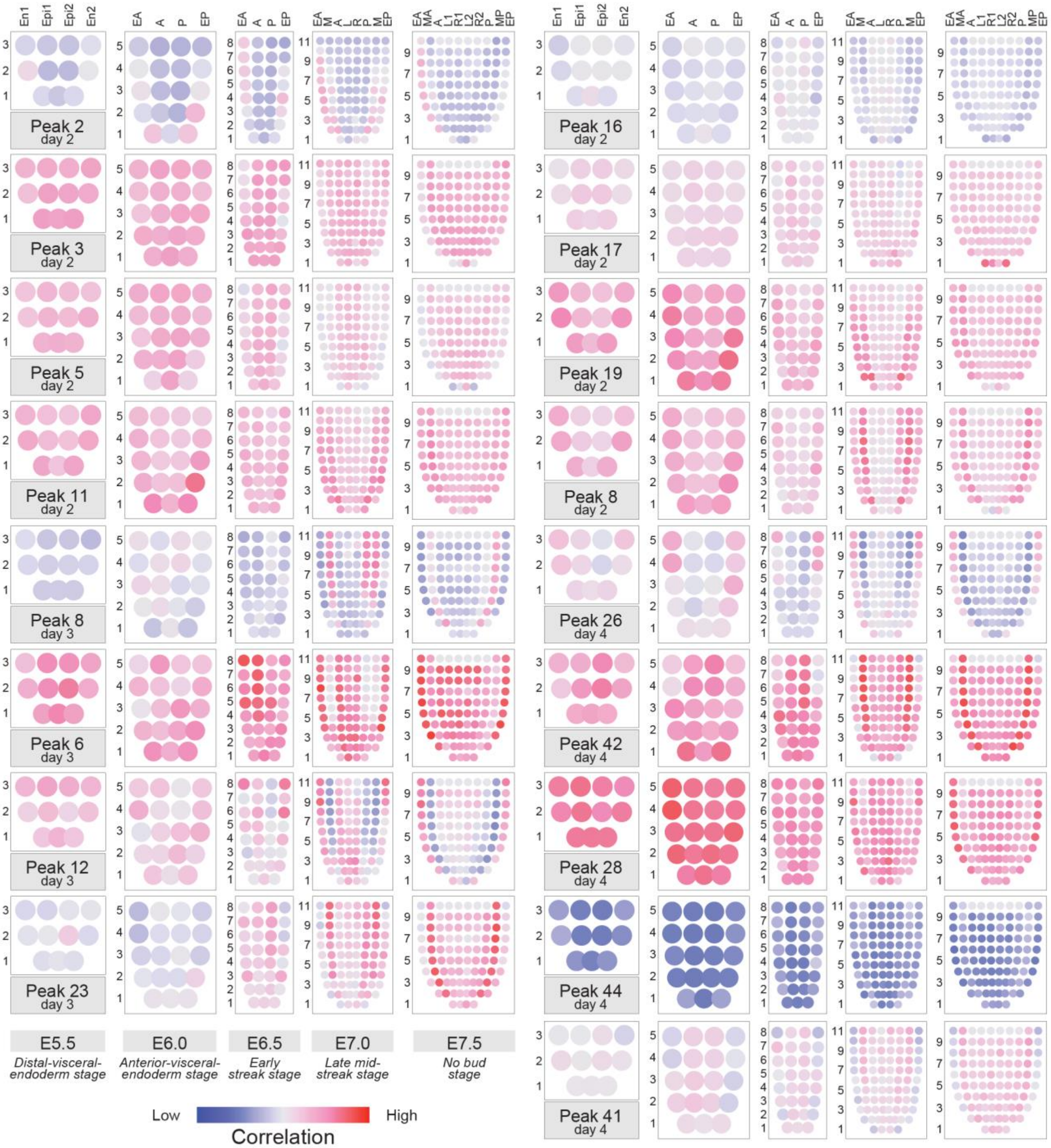

Supplementary Figure 4: Annotation of early TRIAGE cell type peaks with reference to spatial gene expression domains in murine embryogenesis. TRIAGE peaks with cells from days 2-4 of differentiation mapped to developing mouse embryo at five different stages of development between embryonic (E) days 5.5 and 7.5, spanning pre-gastrulation to late primitive streak and amnion formation[8,9]. For each peak and timepoint, the five corn plots capture different stages of development in mouse embryos, where each spot has captured the expression profiles of approximately 5-40 cells. En1 and En2: divided endoderm; Epi1 and Epi2: divided epiblast; EA: anterior endoderm; A: anterior; P: posterior; EP: posterior endoderm; M: whole mesoderm; L: left lateral; R: right lateral; MA: anterior mesoderm; L1: anterior left lateral; R1: anterior right lateral; L2: posterior left lateral; R2: posterior right lateral; MP: posterior mesoderm. See also **Figure 3d** for expert annotation of highly correlated domains for each peak and timepoint.

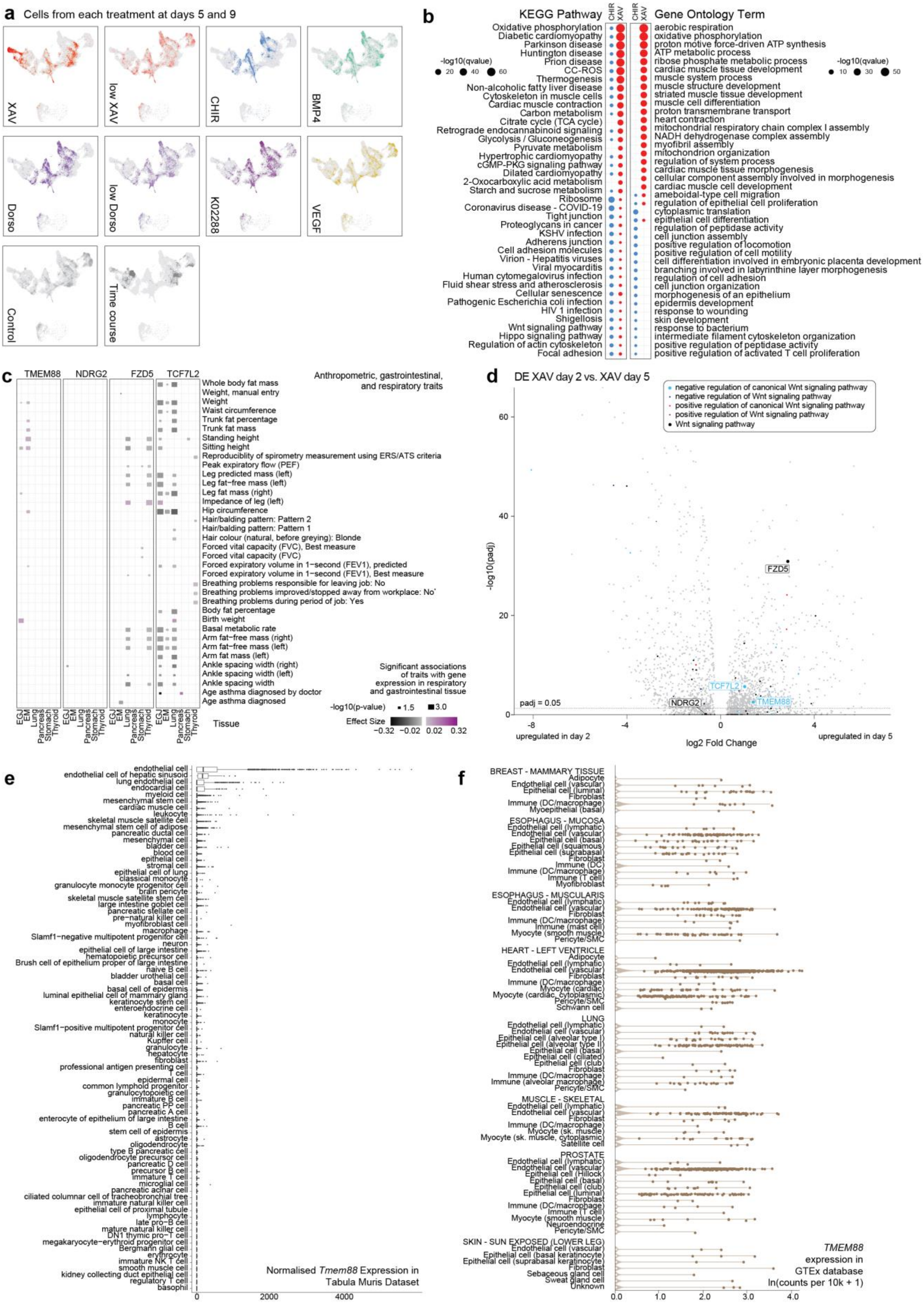

Supplementary Figure 5: Evaluation of Wnt signalling and candidate genes regulating differentiation. a) Cells from each treatment condition allocated to each cell type peak at days 5 and 9 of differentiation. **b)** Enrichment of KEGG

pathways and GO terms in genes that are differentially expressed between XAV and CHIR-treated cells at day 9. CC-ROS: chemical carcinogenesis-reactive oxygen species; KSHV: Kaposi sarcoma-associated herpesvirus; HIV: Human immunodeficiency virus. q-values are derived from p-values from one-sided Fisher's exact test with Benjamini-Hochberg correction for multiple testing. **c)** SMR analysis evaluating associations of candidate WNT-related gene expression in respiratory or gastrointestinal tissues with anthropometric, gastrointestinal, and respiratory traits in the UK Biobank. Some trait and tissue names were shortened from the following: *Breathing problems improved/stopped away from workplace or on holiday: No; EGJ: Esophagus gastroesophageal junction; EM: Esophagus mucosa. P-values were derived from the SMR test statistic (approximate χ^2 statistic). **d)** Differential gene expression analysis comparing XAV-treated cells at days 2 and 5 of differentiation. Genes annotated with the listed GO terms related to the Wnt signalling pathway are coloured and the four candidate Wnt regulators from **Figure 4d-e** are labelled. P-values are determined by Wilcoxon Rank Sum test and corrected for multiple testing using Bonferroni correction with all genes in the dataset. **e)** *Tmem88* gene expression from Tabula Muris dataset[10]. **f)** *TMEM88* gene expression from GTEx[11]. Source data are provided as a Source data file.

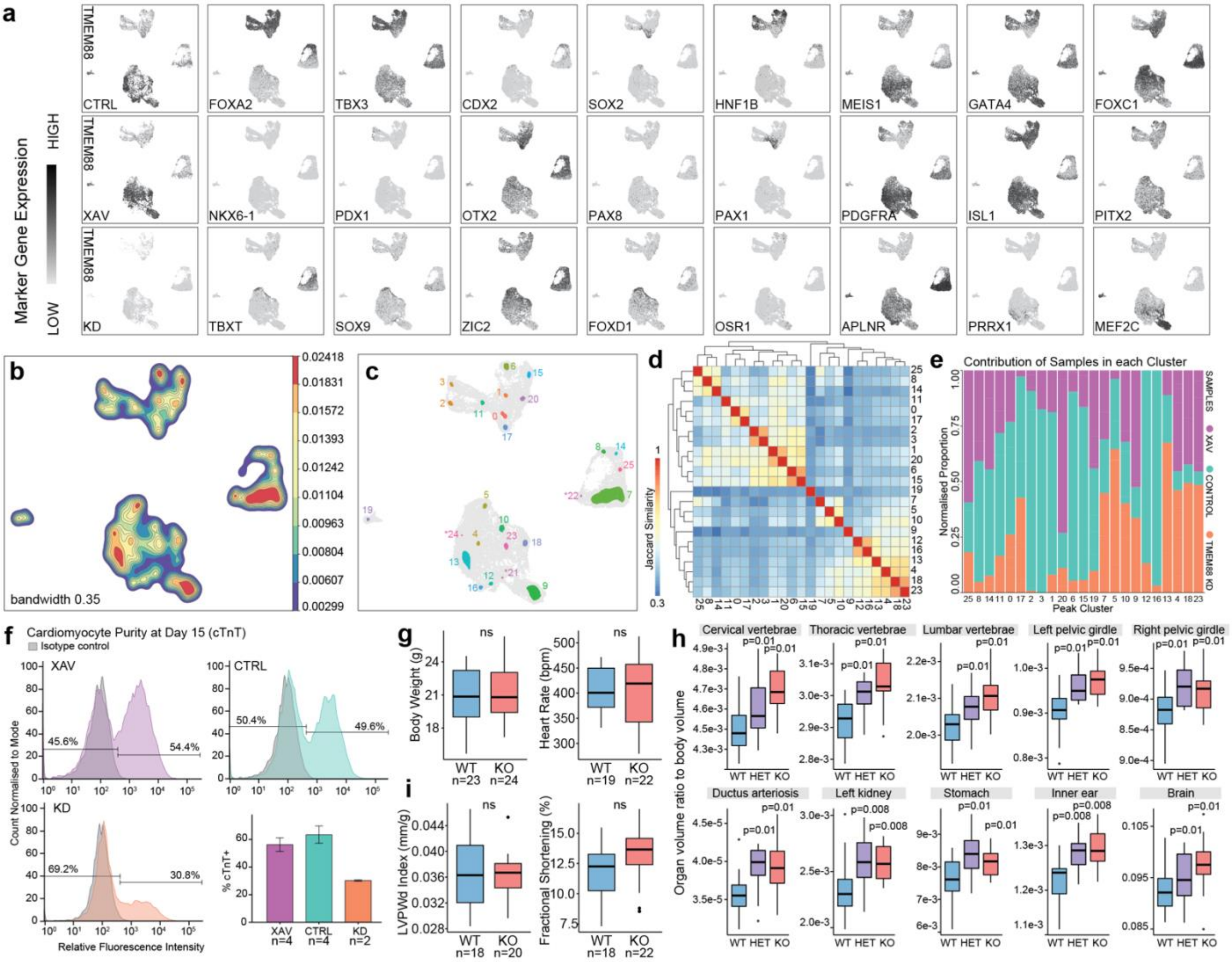

Supplementary Figure 6: *TMEM88* as a regulator of mesendodermal development in hiPSCs and mouse model. **a)** Expression of additional cell type marker genes in the *TMEM88* scRNA-seq dataset. First column shows expression of *TMEM88* compared between the three treatment conditions. *FOXA2* and *TBX3* mark definitive endoderm, *CDX2* marks hindgut, *SOX2* marks pluripotency and anterior foregut, *HNF1B* marks liver progenitors, *MEIS1* and *GATA4* mark mesoderm, *FOXC1* marks somitic mesoderm, *NKX6-1* and *PDX1* mark pancreatic progenitors, *OTX2* marks anterior foregut, *PAX8* marks thyroid progenitors, *PAX1* marks pharyngeal endoderm, *PDGFRA* and *ISL1* mark lateral plate mesoderm, *PITX2* and *PRRX1* mark limb bud mesoderm, *T* and *SOX9* mark axial mesoderm, *ZIC2* and *FOXD1* mark neuromesodermal neural crest progenitors, *OSR1* marks intermediate mesoderm, *ALPNR* marks lateral plate mesoderm, and *MEF2C* marks cardiac mesoderm. **b)** Contour plot showing regions of cells in the UMAP space expressing genes with high repressive tendency scores using bandwidth 0.35 to inform TRIAGE-Cluster[5] analysis. **c)** UMAP showing immediate output from TRIAGE-Cluster, identifying 26 distinct cell types. Cell type labels with an * have fewer than 20 cells and are removed in further analysis. **d)** Heatmap and dendrogram showing pairwise Jaccard similarity between the top 100 genes for each peak after TRIAGE-transformation[6]. **e)** Contribution of cells from each sample to each cell type peak in the dataset. Within each column, proportions sum to 1 and the input number of cells for each sample is normalised to the total number of cells collected in that sample. **f)** Representative plots from FACS analysis for cTnT+ (cardiac troponin T) cells from day 15 of mesendoderm differentiation (left) and summarised FACS results. Data are represented as mean ± standard error. p: p-value, by two-tailed Student's *t*-test; ns: not significant. **g)** Comparison of overall body weight and heart rates between wildtype and *Tmem88* KO mice. ns: not significant by two-

tailed Student's *t*-test. **h)** Summary of micro-computed tomography results showing significant difference in normalised organ volume between heterozygous (HET, n=15) or homozygous (KO, n=17) *Tmem88* mutant E15.5 embryos and wildtype littermates (WT, 20). p: p-value adjusted for multiple comparisons by permutation-based correction to an FDR of 0.01. **i)** Echocardiographic analysis of heart function in *Tmem88* KO vs control (WT). Left ventricular posterior wall at diastole (LVPWd) index normalised to body weight. ns: not significant by two-tailed Student's *t*-test. In box plots, centre line shows median, box limits show upper and lower quartiles, whiskers show 1.5x interquartile range, and points show outliers. Source data are provided as a Source data file.

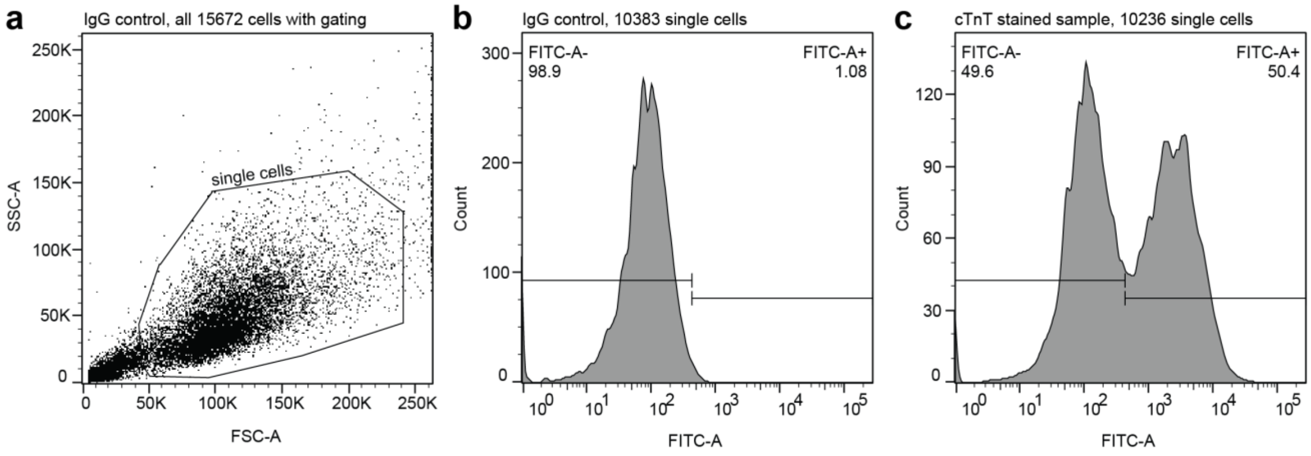

Supplementary Figure 7: Gating strategy used for flow cytometry analysis. a) Gating used to identify starting single cell population in an immunoglobin G (IgG) isotype control. **b)** Gating used on single cell population in the IgG control sample to define upper bound of negative population. **c)** Gating carried over from IgG control in **(b)** for cTnT-stained sample cells.

SUPPLEMENTARY TABLES

Supplementary Table 1: Quality control and barcoding metrics in iPSC pilot scRNA-seq.

Group	Highest BC/HTO	# cells	% high QC cells	Median total RNA reads	Median total genes captured	Median #BC/HTO reads	Median # mapped BC/HTOs	Median 1st BC/HTO Count	Median 2nd BC/HTO Count
All barcoded cells	BC01	421	91.21	21032	4365	16	2	15	1
	BC02	473	89.22	19924	4254	19	1	18	0
	BC03	231	92.64	23127	4707	13	2	12	1
	BC04	265	90.94	22652	4540	10	2	9	1
	BC05	226	88.05	21561	4442	23	2	23	1
	BC06	315	85.40	21130	4379	37	2	36	1
	BC07	226	79.65	24494	4922	25	1	24	0
	BC08	222	79.28	25161	4888	23	2	22	1
	BC09	188	93.09	21355	4395	14	2	14	1
	BC10	184	88.59	21054	4432	20	2	19	1
	BC11	381	87.14	21669	4391	22	2	21	1
	BC12	188	83.51	24205	4721	23	2	22	1
	BC13	186	82.26	24114	4690	20	2	18	1
	BC14	325	92.31	21185	4423	19	2	18	1
	BC15	448	90.85	19942	4184	25	2	23	1
	BC16	339	92.33	20310	4190	30	1	29	0
	BC17	379	89.71	18206	4039	26	2	25	1
	BC18	332	88.25	19199	4121	28	1	27	0
	No BC	1299	4.93	1017	119	0	0	0	0
	Doublet*	83	56.63	15696	3796	2	2	1	1
	All cells	6711	71.96	18944	4121	15	1	14	0
All hashed cells	HTO-A2051 (BC07)	209	85.17	24059	4773	1	1	1	0
	HTO-A2052 (BC08)	160	91.25	25880	4913	1	1	1	0
	HTO-A2053 (BC12)	251	88.84	23105	4610	1	1	1	0
	HTO-A2054 (BC13)	259	91.89	23697	4701	1	1	1	0
	HTO Doublet*	45	100.00	30328	5217	2	2	1	1
	All BC + HTO	924	89.83	24066	4733	1	1	1	0
Barcoded cells negative for hashing oligos	BC07 (No HTO)	93	79.57	22983	4814	24	1	22	0
	BC08 (No HTO)	141	70.92	22470	4658	19	1	18	0
	BC12 (No HTO)	78	76.92	23259	4632	19	1	18	0
	BC13 (No HTO)	95	76.84	21018	4426	17	2	16	1
	BC07,08,12,13 (No HTO)	407	75.43	22367	4623	19	1	18	0
Hashed cells negative for barcodes	HTO-A2051 (No BC)	68	4.41	1312	108	1	1	1	0
	HTO-A2052 (No BC)	41	2.44	1151	113	1	1	1	0
	HTO-A2053 (No BC)	45	2.22	1066	106	1	1	1	0
	HTO-A2054 (No BC)	25	8.00	1273	148	1	1	1	0
	HTO Doublet (No BC)*	1	0.00	1279	216	1	2	1	1
	All HTO only (no BC)	180	3.89	1227	113	2	1	1	0

*Doublet cells refer to cells with exactly the same number of reads mapped to >1 barcode or HTO. QC: Quality Control; BC: genomic barcode; HTO: cell hashing oligonucleotide.

Supplementary Table 2: Quality control and barcoding metrics in atlas of differentiation dataset.

Sample/Library	Cell Count (Pre-filter)	Median Library Size	Median # Genes	Median % Mt	Median % Rb	Median BC/HTO reads	Median BC/HTOs captured	Median %reads for 1st BC/HTO	Median %reads for 2nd BC/HTO	% Negative BC/HTO	% Doublet BC/HTO	% Singlet BC/HTO	Final Cell Count (post QC)
**Time Course (HTO)**	19997	23575	4984	5.83	27.93	605	7	87.53	1.36	1.75	20.09	78.17	13682
**Signalling Lib 1 (BC)**	19656	22812	4926	6.89	29.15	204	18	51.77	6.44	2.17	23.83	74.00	16586
**Signalling Lib 2 (BC)**	19708	21801	4761	8.06	27.29	225	17	56.14	5.73	0.13	22.42	77.45	16391
**Signalling Lib 3 (BC)**	19349	21063	4992	5.86	21.63	142	17	51.75	6.40	10.42	23.83	65.75	15549
**Control Day 2 (HTO-A0252)**	531	23483	4768	8.38	28.74	541	8	88.25	1.44	0.19	20.53	79.28	330
**Control Day 2 (Lib 2, BC01)**	1664	22108	4517.5	8.14	33.86	215	17	56.26	5.64	0.06	15.02	84.92	1635
**Control Day 2 (Lib 1, BC10)**	1003	28483	5264	7.72	32.01	248	18	52.90	6.15	0.90	31.21	67.90	934
**Control Day 5 (HTO-A0255)**	3192	18284.5	4228	3.57	34.34	315	7	79.68	2.17	8.49	12.91	78.60	2687
**Control Day 5 (Lib 1, BC01)**	1203	22137	4906	5.57	29.77	223	18	58.91	5.41	2.08	21.11	76.81	1142
**Control Day 5 (Lib 3, BC10)**	1304	22574	5184	6.01	22.42	168.5	18	56.67	5.59	7.29	25.54	67.18	1148
**Control Day 9 (HTO-A0251)**	744	21076.5	4674.5	5.61	25.07	887	8	91.99	1.10	0.13	27.82	72.04	605
**Control Day 9 (Lib 3, BC01)**	1165	22433	4913	5.47	25.20	162	18	55.89	5.77	7.38	26.27	66.35	1017
**Control Day 9 (Lib2, BC10)**	1265	19625	4705	6.85	24.66	222	17	60.18	5.37	0.16	23.79	76.05	1218

SUPPLEMENTARY REFERENCES

1. Pijuan-Sala, B., Griffiths, J.A., Guibentif, C., Hiscock, T.W., Jawaid, W., Calero-Nieto, F.J., Mulas, C., Ibarra-Soria, X., Tyser, R.C.V., Ho, D.L.L., et al. (2019). A single-cell molecular map of mouse gastrulation and early organogenesis. Nature *566*, 490-495. 10.1038/s41586-019-0933-9.

2. Qiu, C., Martin, B.K., Welsh, I.C., Daza, R.M., Le, T.-M., Huang, X., Nichols, E.K., Taylor, M.L., Fulton, O., O'Day, D.R., et al. (2024). A single-cell time-lapse of mouse prenatal development from gastrula to birth. Nature *626*, 1084-1093. 10.1038/s41586-024-07069-w.

3. Tyser, R.C.V., Mahammadov, E., Nakanoh, S., Vallier, L., Scialdone, A., and Srinivas, S. (2021). Single-cell transcriptomic characterization of a gastrulating human embryo. Nature *600*, 285-289. 10.1038/s41586-021-04158-y.

4. Farrell, J.A., Wang, Y., Riesenfeld, S.J., Shekhar, K., Regev, A., and Schier, A.F. (2018). Single-cell reconstruction of developmental trajectories during zebrafish embryogenesis. Science *360*. 10.1126/science.aar3131.

5. Sun, Y., Shim, W.J., Shen, S., Sinniah, E., Pham, D., Su, Z., Mizikovsky, D., White, M.D., Ho, Joshua W.K., Nguyen, Q., et al. (2023). Inferring cell diversity in single cell data using consortium-scale epigenetic data as a biological anchor for cell identity. Nucleic Acids Research, gkad307. 10.1093/nar/gkad307.

6. Shim, W.J., Sinniah, E., Xu, J., Vitrinel, B., Alexanian, M., Andreoletti, G., Shen, S., Sun, Y., Balderson, B., Boix, C., et al. (2020). Conserved Epigenetic Regulatory Logic Infers Genes Governing Cell Identity. Cell Systems. 10.1016/j.cels.2020.11.001.

7. Hutchins, A.P., Yang, Z., Li, Y., He, F., Fu, X., Wang, X., Li, D., Liu, K., He, J., Wang, Y., et al. (2017). Models of global gene expression define major domains of cell type and tissue identity. Nucleic Acids Research *45*, 2354-2367. 10.1093/nar/gkx054.

8. Peng, G., Suo, S., Cui, G., Yu, F., Wang, R., Chen, J., Chen, S., Liu, Z., Chen, G., Qian, Y., et al. (2019). Molecular architecture of lineage allocation and tissue organization in early mouse embryo. Nature *572*, 528-532. 10.1038/s41586-019-1469-8.

9. Rivera-Pérez, J.A., Jones, V., and Tam, P.P.L. (2010). Chapter 11 - Culture of Whole Mouse Embryos at Early Postimplantation to Organogenesis Stages: Developmental Staging and Methods. In Methods in Enzymology, P.M. Wassarman, and P.M. Soriano, eds. (Academic Press), pp. 185-203. https://doi.org/10.1016/S0076-6879(10)76011-0.

10. The Tabula Muris Consortium, Schaum, N., Karkanias, J., Neff, N.F., May, A.P., Quake, S.R., Wyss-Coray, T., Darmanis, S., Batson, J., Botvinnik, O., et al. (2018). Single-cell transcriptomics of 20 mouse organs creates a Tabula Muris. Nature *562*, 367-372. 10.1038/s41586-018-0590-4.

11. GTEx (2020). The GTEx Consortium atlas of genetic regulatory effects across human tissues. Science *369*, 1318. 10.1126/science.aaz1776.
