# Supplementary material for: Atlas of multilineage stem cell differentiation reveals TMEM88 as a developmental regulator of blood pressure
Source: Nat Commun. 2025 Feb 4;16:1356. doi: 10.1038/s41467-025-56533-2 (PMC11794859; doi:10.1038/s41467-025-56533-2)
Supplement: Supplementary file 3 — Description of Additional Supplementary Files [file 41467_2025_56533_MOESM3_ESM.pdf]

Description of Additional Supplementary Files

Supplementary Data 1: Barcoding plasmid DNA sequence.

Supplementary Data 2: All CellChat significant ligand-receptor interactions. P-values for interactions are derived

using a permutation test.

Supplementary Data 3: CellphoneDB cell-cell interaction enrichment tables.

Supplementary Data 4: Enrichment of all regulons' activity in each cluster from pySCENIC analysis.

Supplementary Data 5: TRIAGE genes used for identifying each cell type peak in the atlas dataset.

Supplementary Data 6: Top 100 TRIAGE identity-defining genes for each TRIAGE-Cluster cell type peak and their

TRIAGE-ParseR gene cluster assignments in the atlas dataset.

Supplementary Data 7: Specificity of cell identity-defining TRIAGE-ParseR genes across all TRIAGE-Cluster cell

type peaks in the atlas dataset.

Supplementary Data 8: GO terms enriched for each TRIAGE-ParseR gene cluster in each TRIAGE-Cluster cell type

peak in the atlas dataset. Enrichment p-values determined by a one-tailed hypergeometric test and adjusted using

Benjamini-Hochberg FDR correction).

Supplementary Data 9: SMR analysis results for the top 100 most enriched genes as well as all differentially Wntrelated genes' association to systolic and diastolic blood pressure in cardiovascular tissue.
