# Supplementary figures and images for: Atlas of multilineage stem cell differentiation reveals TMEM88 as a developmental regulator of blood pressure
Source: Nat Commun. 2025 Feb 4;16:1356. doi: 10.1038/s41467-025-56533-2 (PMC11794859; doi:10.1038/s41467-025-56533-2)

# Sum of significant interactions

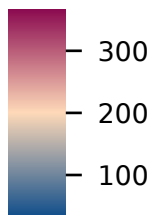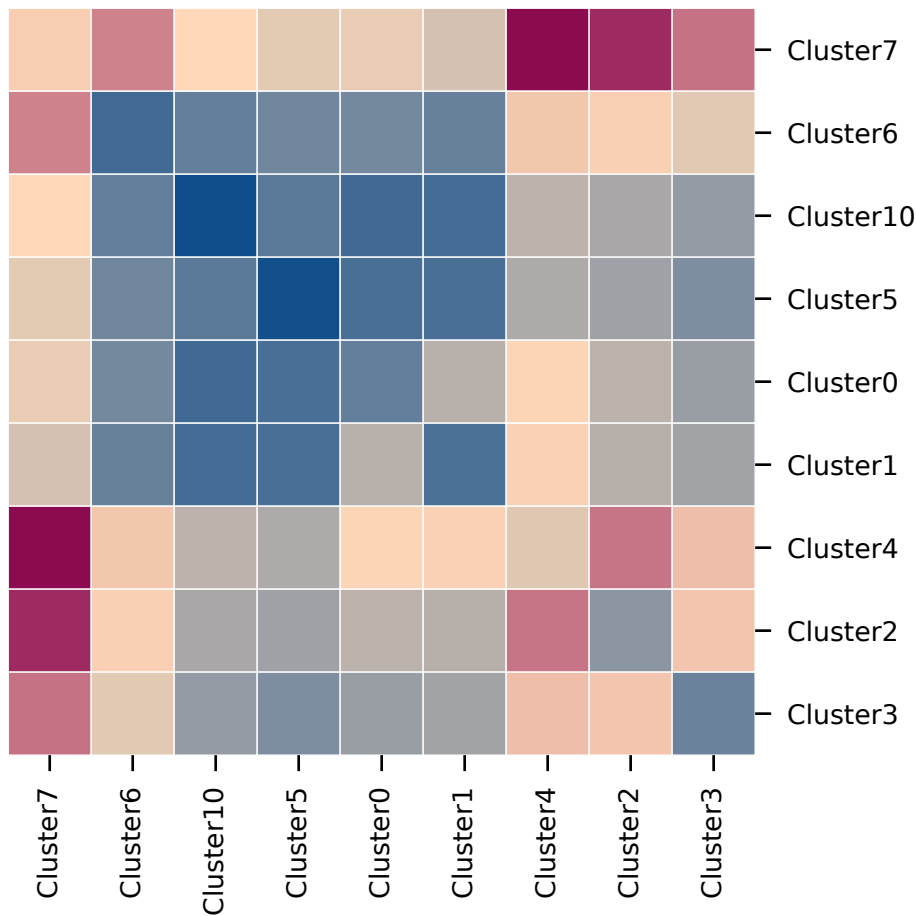

Supplement: Supplementary file 6 — Supplementary Data 3 [file 41467_2025_56533_MOESM6_ESM.zip › cpdb_results/no_treatment_day2_method2/heatmap.pdf]

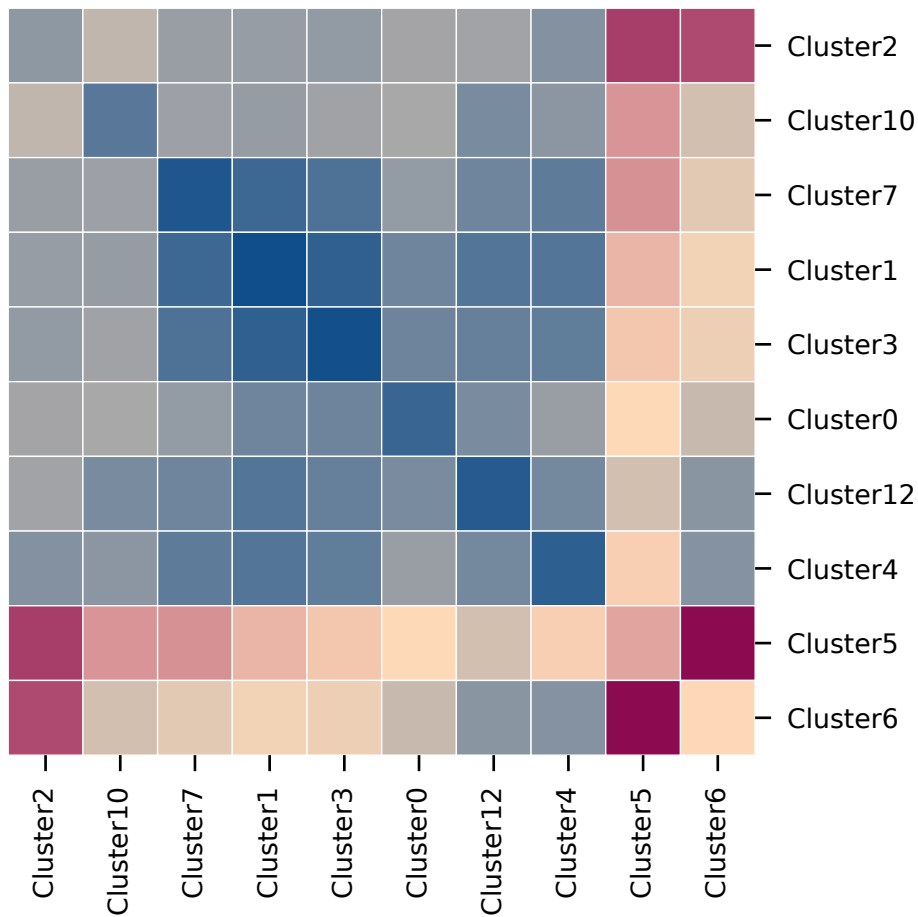

Supplement: Supplementary file 6 — Supplementary Data 3 [file 41467_2025_56533_MOESM6_ESM.zip › cpdb_results/no_treatment_tc_day2_method2/heatmap.pdf]

Sum of significant interactions

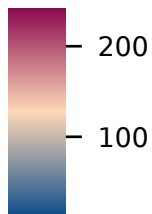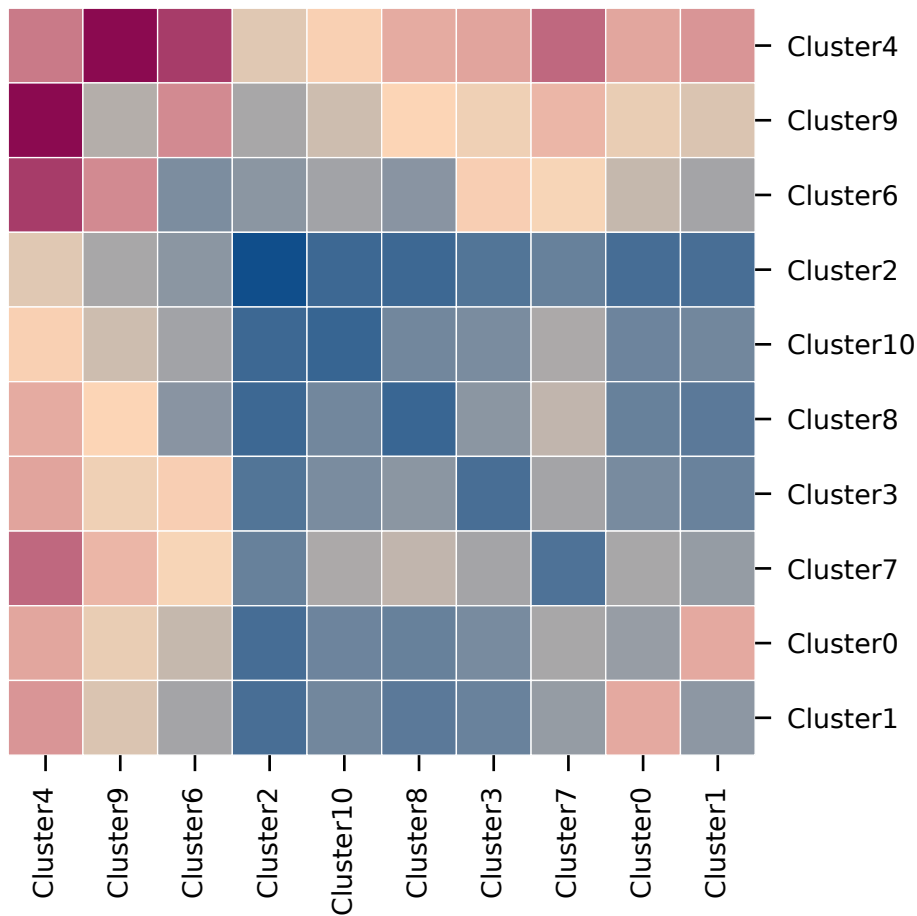

Supplement: Supplementary file 6 — Supplementary Data 3 [file 41467_2025_56533_MOESM6_ESM.zip › cpdb_results/lowDorso_day2_method2/heatmap.pdf]

Sum of significant interactions

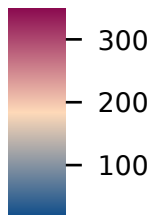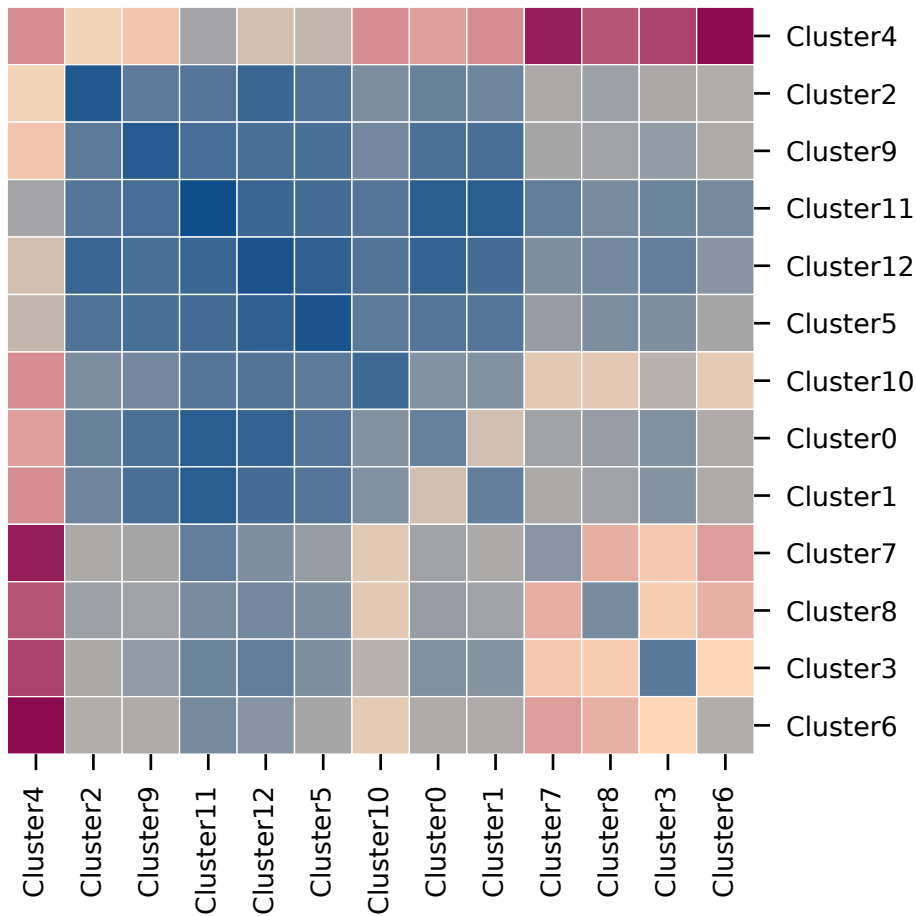

Supplement: Supplementary file 6 — Supplementary Data 3 [file 41467_2025_56533_MOESM6_ESM.zip › cpdb_results/lowXAV_day2_method2/heatmap.pdf]

Sum of significant interactions

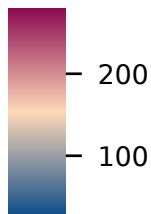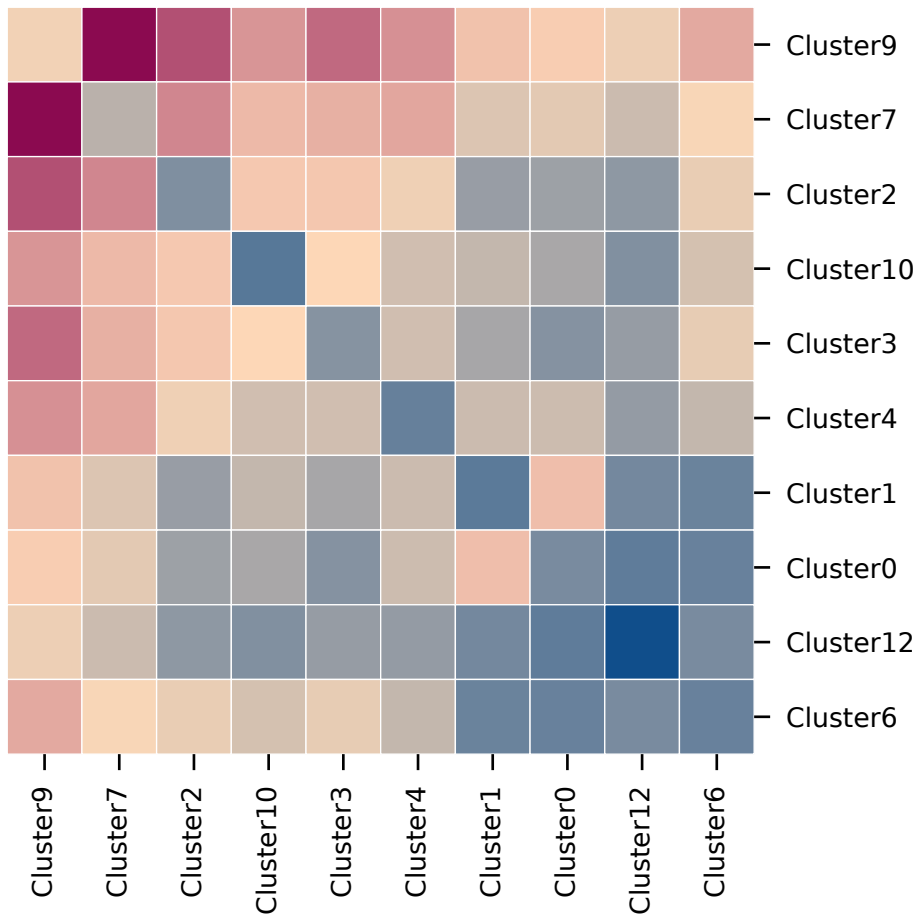

Supplement: Supplementary file 6 — Supplementary Data 3 [file 41467_2025_56533_MOESM6_ESM.zip › cpdb_results/Dorso_day2_method2/heatmap.pdf]

Sum of significant interactions

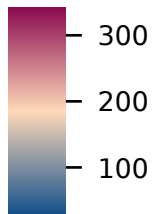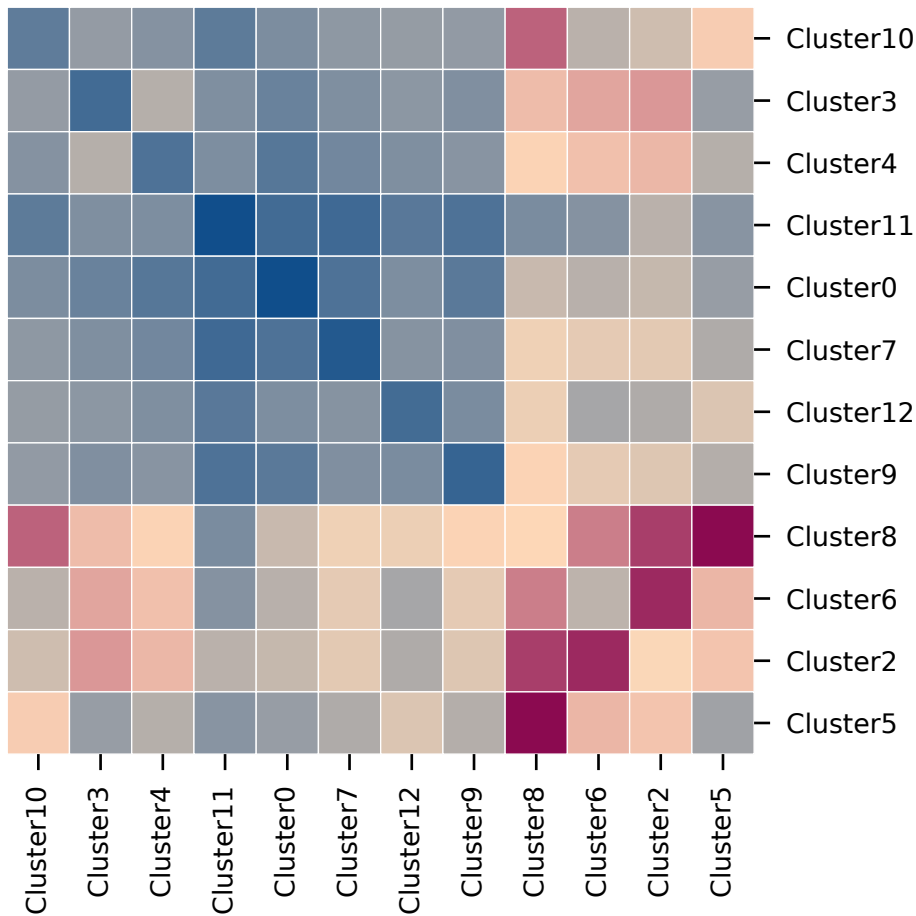

Supplement: Supplementary file 6 — Supplementary Data 3 [file 41467_2025_56533_MOESM6_ESM.zip › cpdb_results/XAV_day5_method2/heatmap.pdf]

Sum of significant interactions

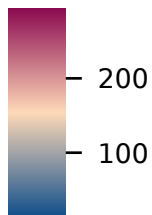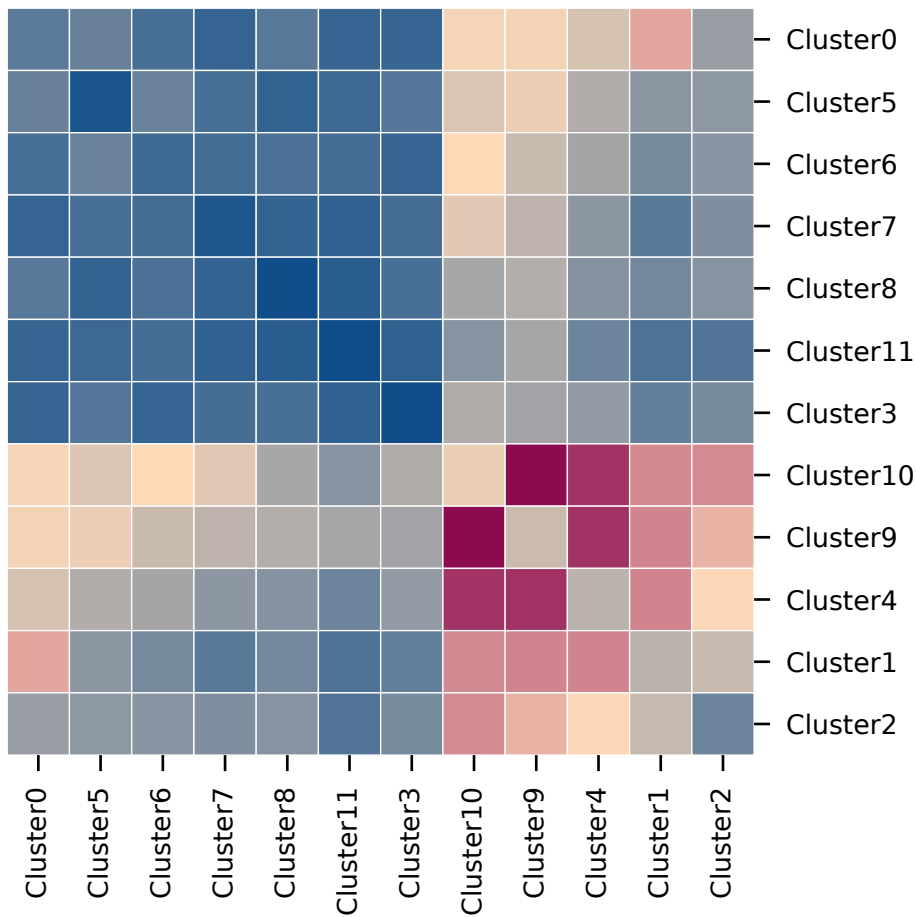

Supplement: Supplementary file 6 — Supplementary Data 3 [file 41467_2025_56533_MOESM6_ESM.zip › cpdb_results/CHIR_day2_method2/heatmap.pdf]

Sum of significant interactions

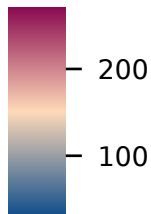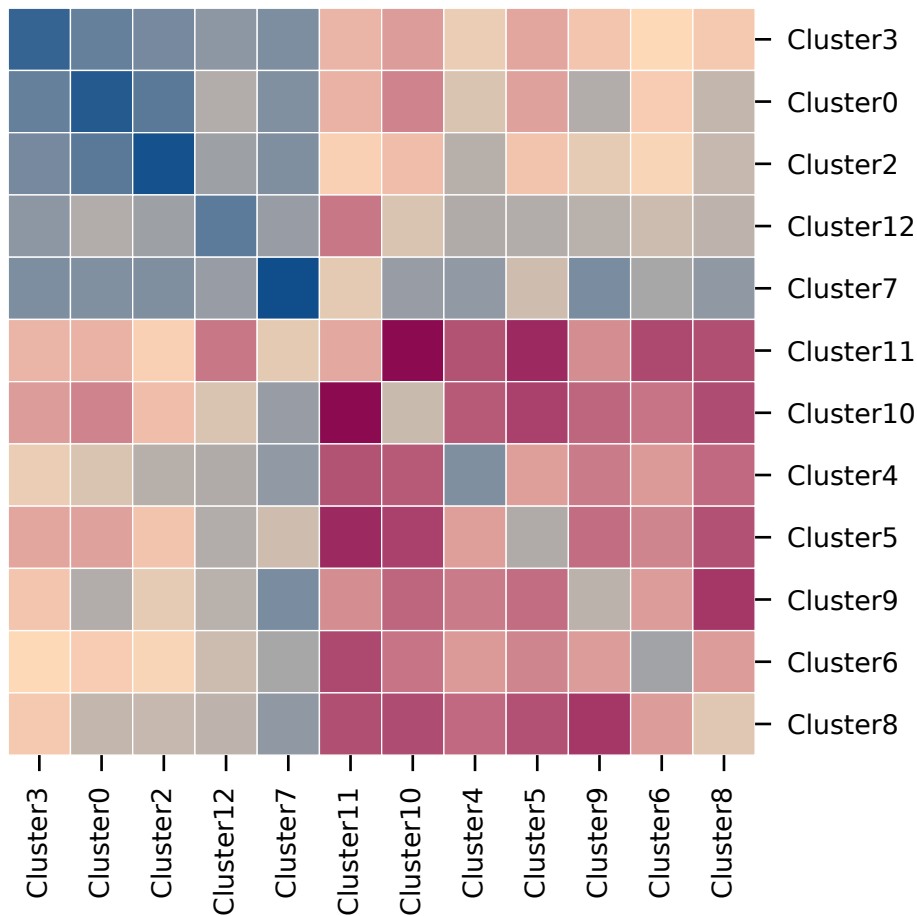

Supplement: Supplementary file 6 — Supplementary Data 3 [file 41467_2025_56533_MOESM6_ESM.zip › cpdb_results/BMP4_day9_method2/heatmap.pdf]

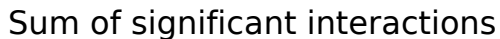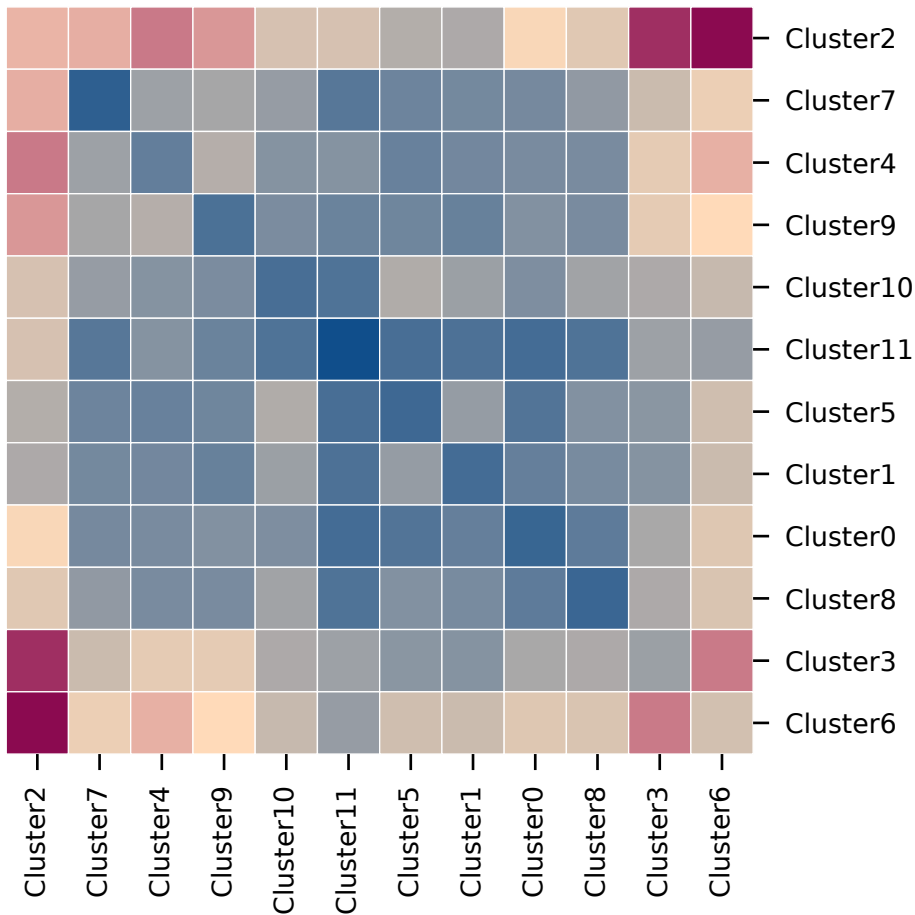

Supplement: Supplementary file 6 — Supplementary Data 3 [file 41467_2025_56533_MOESM6_ESM.zip › cpdb_results/lowXAV_day5_method2/heatmap.pdf]

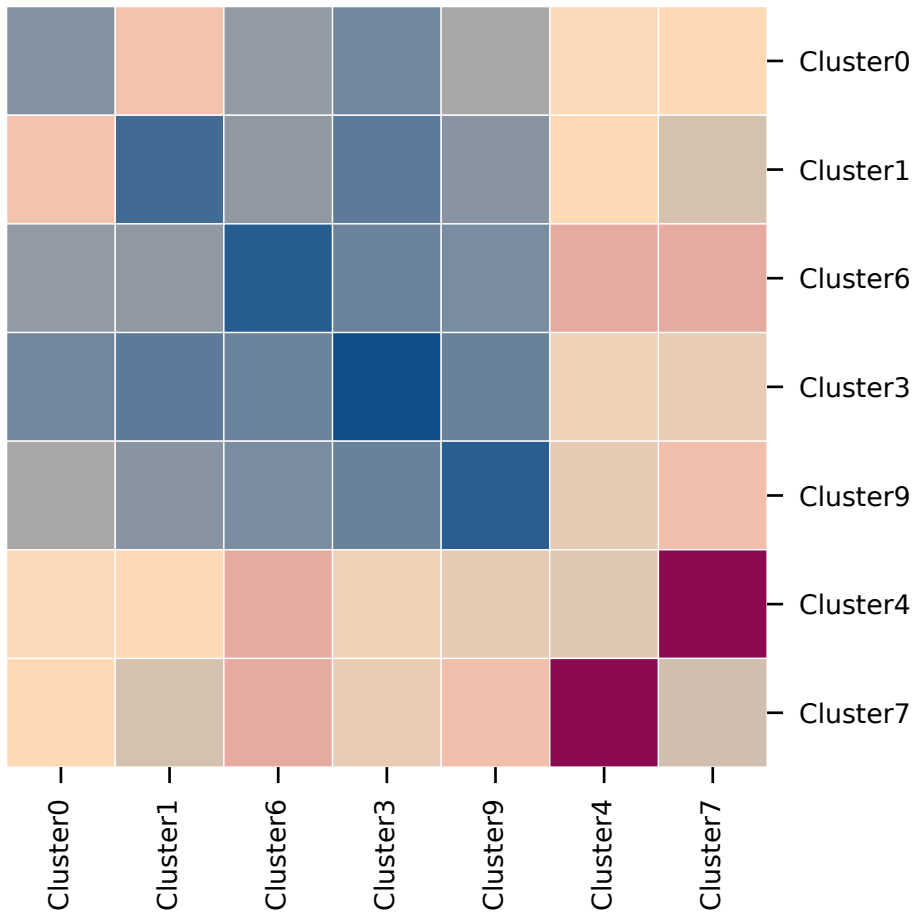

Supplement: Supplementary file 6 — Supplementary Data 3 [file 41467_2025_56533_MOESM6_ESM.zip › cpdb_results/XAV_day2_method2/heatmap.pdf]

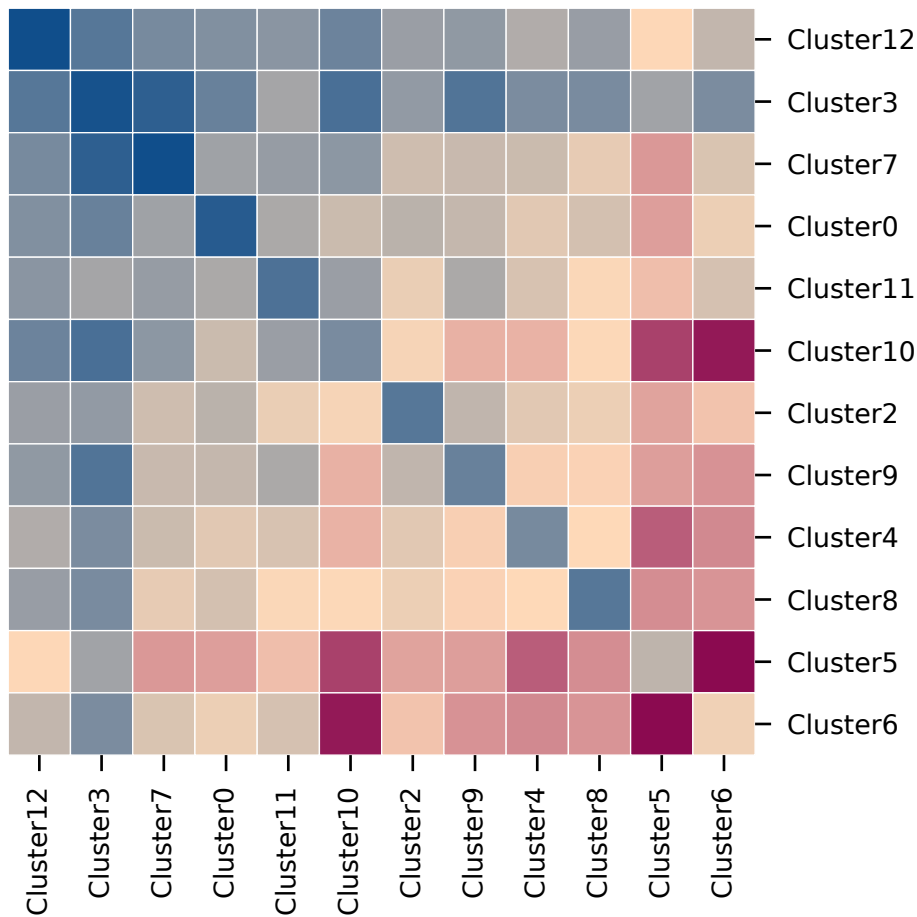

Supplement: Supplementary file 6 — Supplementary Data 3 [file 41467_2025_56533_MOESM6_ESM.zip › cpdb_results/XAV_day9_method2/heatmap.pdf]

Sum of significant interactions

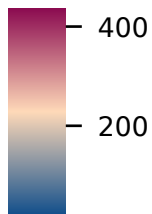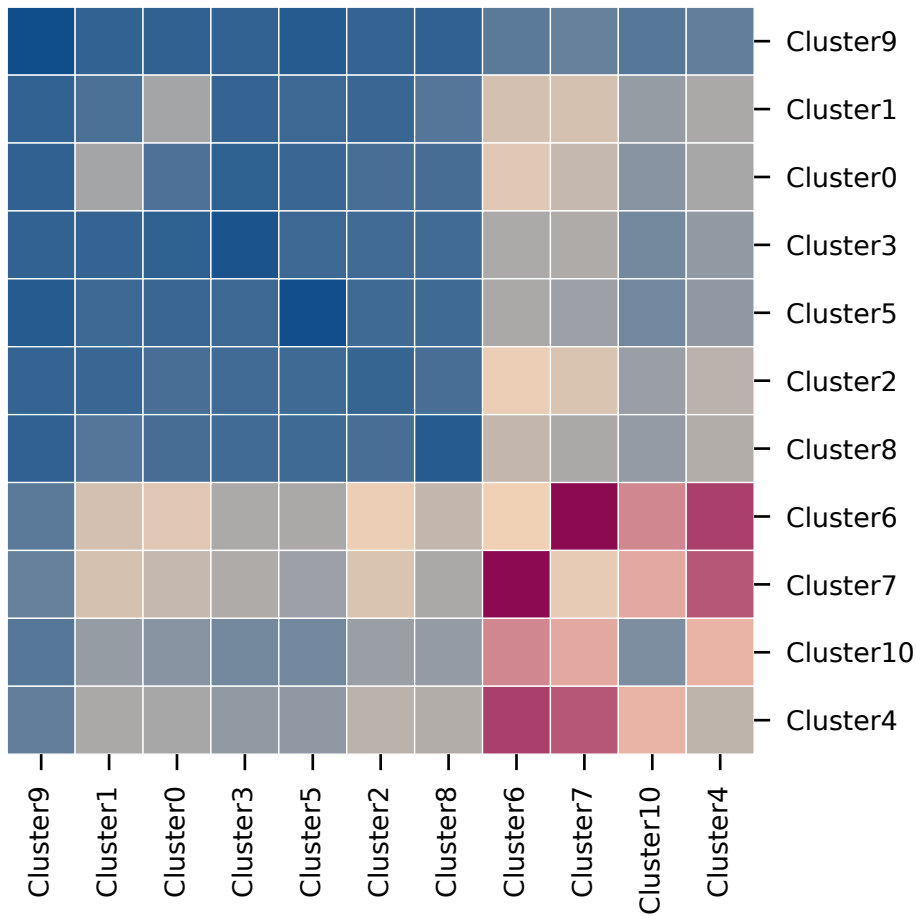

Supplement: Supplementary file 6 — Supplementary Data 3 [file 41467_2025_56533_MOESM6_ESM.zip › cpdb_results/VEGF_day2_method2/heatmap.pdf]

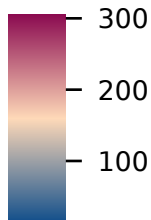

Sum of significant interactions

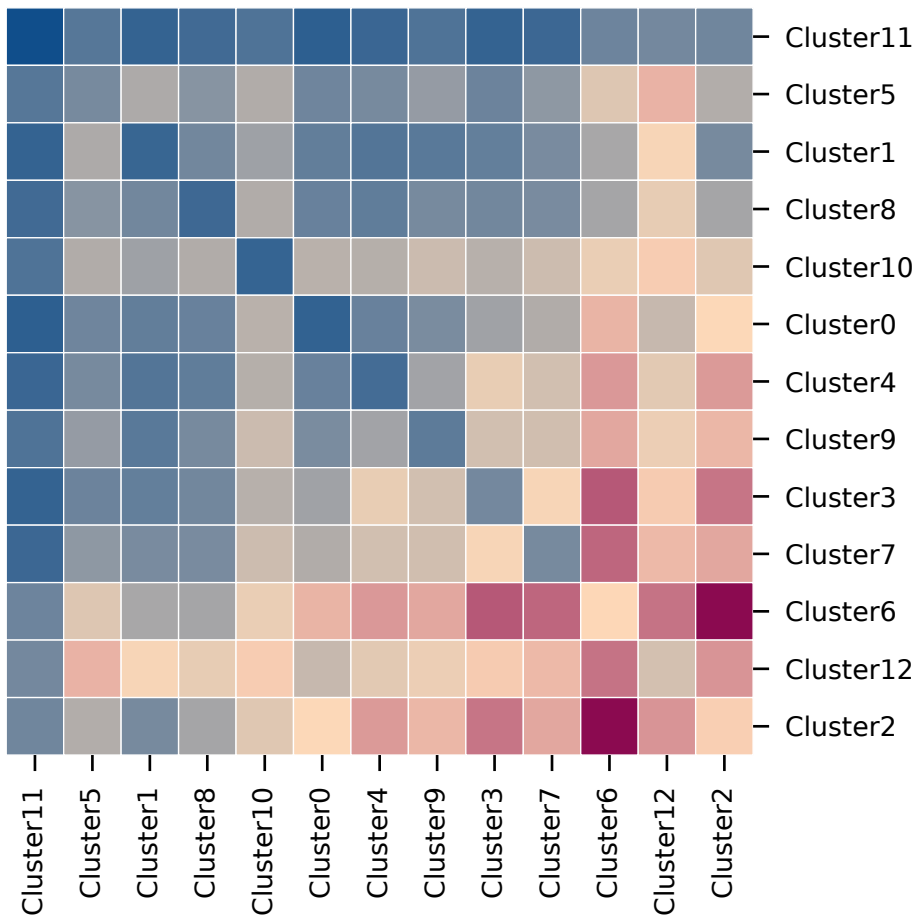

Supplement: Supplementary file 6 — Supplementary Data 3 [file 41467_2025_56533_MOESM6_ESM.zip › cpdb_results/BMP4_day5_method2/heatmap.pdf]

# Sum of significant interactions

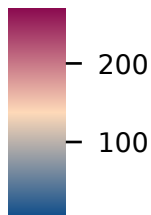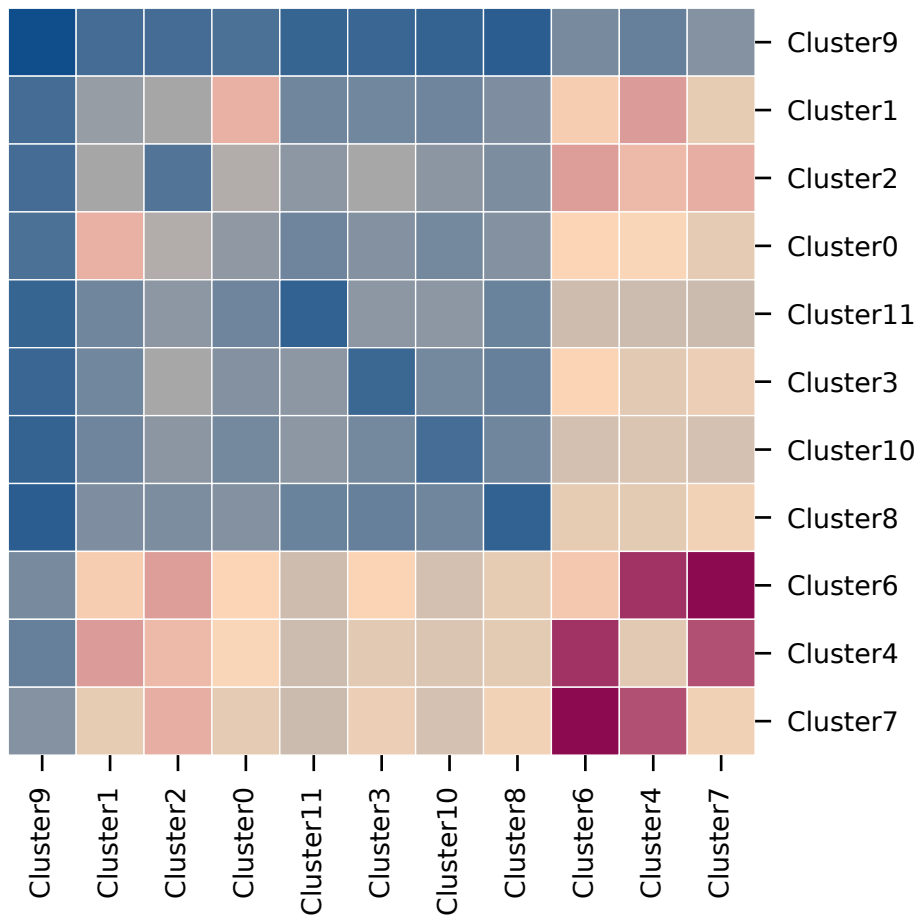

Supplement: Supplementary file 6 — Supplementary Data 3 [file 41467_2025_56533_MOESM6_ESM.zip › cpdb_results/BMP4_day2_method2/heatmap.pdf]

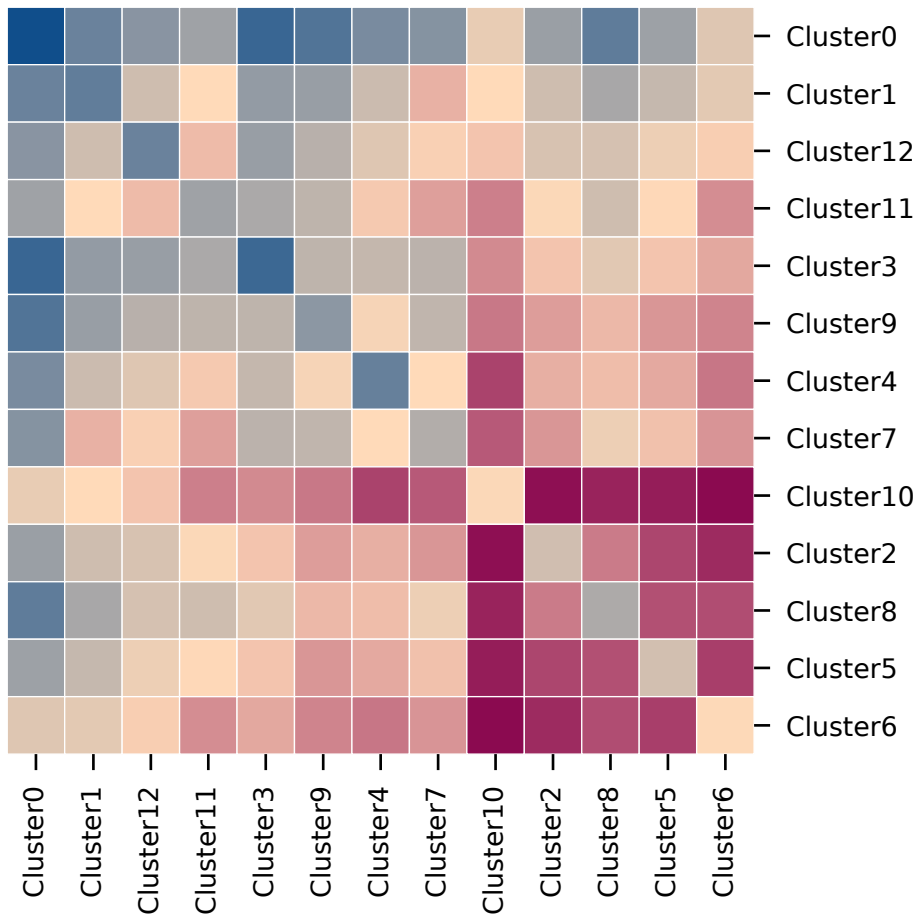

Supplement: Supplementary file 6 — Supplementary Data 3 [file 41467_2025_56533_MOESM6_ESM.zip › cpdb_results/lowXAV_day9_method2/heatmap.pdf]

Sum of significant interactions

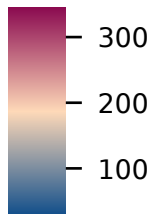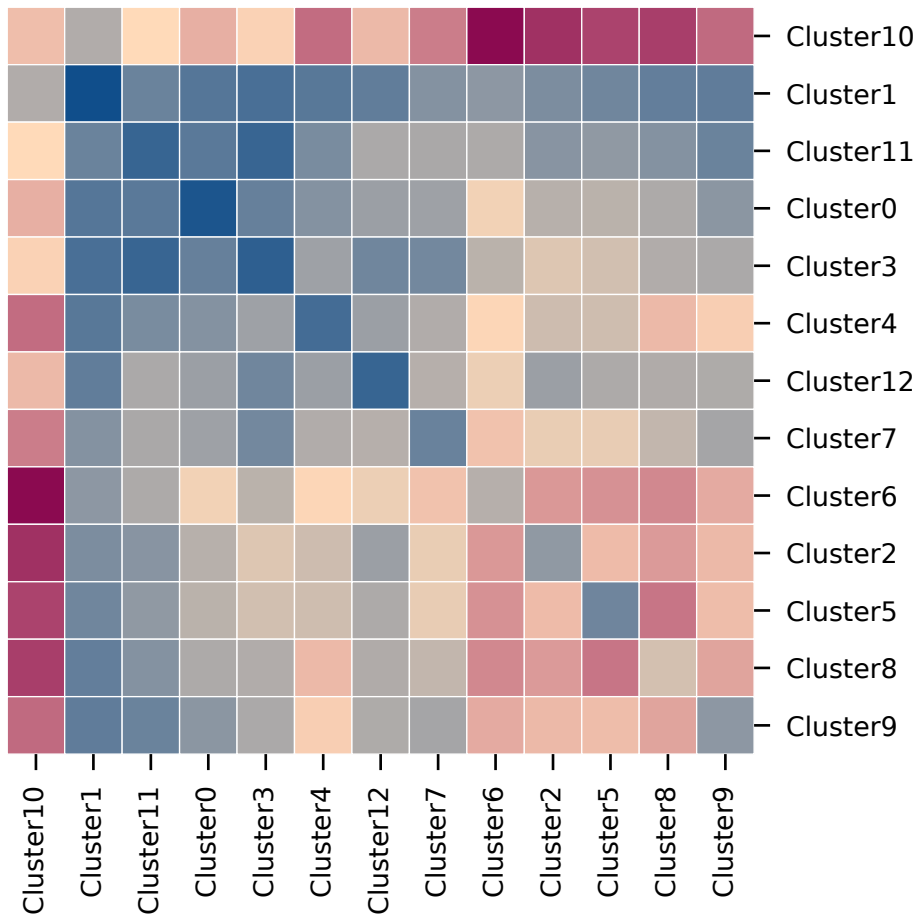

Supplement: Supplementary file 6 — Supplementary Data 3 [file 41467_2025_56533_MOESM6_ESM.zip › cpdb_results/K02288_day9_method2/heatmap.pdf]
