# Supplementary material for: Atlas of multilineage stem cell differentiation reveals TMEM88 as a developmental regulator of blood pressure
Source: Nat Commun. 2025 Feb 4;16:1356. doi: 10.1038/s41467-025-56533-2 (PMC11794859; doi:10.1038/s41467-025-56533-2)
Supplement: Supplementary file 13 — Reporting Summary [file 41467_2025_56533_MOESM13_ESM.pdf]

nature portfolio

Corresponding author(s): A/Prof Nathan Palpant

Last updated by author(s): Dec 2, 2024

Reporting Summary

Nature Portfolio wishes to improve the reproducibility of the work that we publish. This form provides structure for consistency and transparency in reporting. For further information on Nature Portfolio policies, see our Editorial Policies and the Editorial Policy Checklist.

Statistics

For all statistical analyses, confirm that the following items are present in the figure legend, table legend, main text, or Methods section.

n/a	Confirmed	
☐	☒	The exact sample size (*n*) for each experimental group/condition, given as a discrete number and unit of measurement
☐	☒	A statement on whether measurements were taken from distinct samples or whether the same sample was measured repeatedly
☐	☒	The statistical test(s) used AND whether they are one- or two-sided<br>*Only common tests should be described solely by name; describe more complex techniques in the Methods section.*
☒	☐	A description of all covariates tested
☐	☒	A description of any assumptions or corrections, such as tests of normality and adjustment for multiple comparisons
☐	☒	A full description of the statistical parameters including central tendency (e.g. means) or other basic estimates (e.g. regression coefficient) AND variation (e.g. standard deviation) or associated estimates of uncertainty (e.g. confidence intervals)
☐	☒	For null hypothesis testing, the test statistic (e.g. *F*, *t*, *r*) with confidence intervals, effect sizes, degrees of freedom and *P* value noted<br>*Give P values as exact values whenever suitable.*
☒	☐	For Bayesian analysis, information on the choice of priors and Markov chain Monte Carlo settings
☒	☐	For hierarchical and complex designs, identification of the appropriate level for tests and full reporting of outcomes
☒	☐	Estimates of effect sizes (e.g. Cohen's *d*, Pearson's *r*), indicating how they were calculated

Our web collection on statistics for biologists contains articles on many of the points above.

Software and code

Policy information about availability of computer code

Data collection	NovaSeq control software (v1.6.0)<br>Real Time Analysis (v3.4.4)<br>BD FACSDiva (v9.0)<br>Vevo 3100 (v3.8.2)<br>LabChart Pro (v8)
Data analysis	10x Genomics Cellranger (v3.0.2 & v.3.1.0)<br>CITE-seq-Count (v4.2.1)<br>R (v3.6.0 & v4.2.1)<br>scds (v1.2.0)<br>Seurat (v4.0)<br>Nebulosa (v0.99.92)<br>RISC (v1.0)<br>CellChat (v2.1.2)<br>CellPhoneDB (v5.0.0)<br>cellphonedb-data (v5.0)<br>pySCENIC (v0.12.1)<br>URD (v1.1.1)<br>scipy (v1.7.1)<br>sklearn (v0.22)<br>biomaRt (v2.46.3)

TRIAGE-Cluster (https://github.com/palpant-comp/TRIAGE-Cluster)
biomaRt (v2.46.3)
TRIAGE (https://github.com/palpant-comp/TRIAGE)
Cepo (v1.12.0)
pheatmap (v1.0.12)
TRIAGE-ParseR (https://github.com/palpant-comp/TRIAGE-ParseR)
clusterProfiler (v4.6.2)
FlowJo (v10.07)
GraphPad Prism (v7.04)
VevoLab (v3.1.1)
ggpubr (v0.2.3)

For manuscripts utilizing custom algorithms or software that are central to the research but not yet described in published literature, software must be made available to editors and reviewers. We strongly encourage code deposition in a community repository (e.g. GitHub). See the Nature Portfolio guidelines for submitting code & software for further information.

Data

Policy information about availability of data

All manuscripts must include a data availability statement. This statement should provide the following information, where applicable:

- Accession codes, unique identifiers, or web links for publicly available datasets
- A description of any restrictions on data availability
- For clinical datasets or third party data, please ensure that the statement adheres to our policy

All raw and processed single-cell RNA sequencing data generated in this study have been deposited in the NCBI Gene Expression Omnibus repository (GSE279710 [https://www.ncbi.nlm.nih.gov/geo/query/acc.cgi?acc=GSE279710]). An interactive version of the combined, annotated dataset is also available at http://cellfateexplorer.d24h.hk/, where additional TRIAGE-ParseR analysis outputs, metadata, and gene expression for individual cell type peaks can be queried. Source data are provided with this paper. Published datasets used as references include mouse organogenesis data (E-MTAB-6967 [http://www.ebi.ac.uk/microarray-as/aer/result?queryFor=Experiment&eAccession=E-MTAB-6967]); human gastrulation data (E-MTAB-9388 [http://www.ebi.ac.uk/microarray-as/aer/result?queryFor=Experiment&eAccession=E-MTAB-9388]); and mouse gastrula development data (GSE186069 [https://www.ncbi.nlm.nih.gov/geo/query/acc.cgi?acc=GSE186069]).

Research involving human participants, their data, or biological material

Policy information about studies with human participants or human data. See also policy information about sex, gender (identity/presentation), and sexual orientation and race, ethnicity and racism.

Reporting on sex and gender	No work involving human participants or their data or biological material.
Reporting on race, ethnicity, or other socially relevant groupings	No work involving human participants or their data or biological material.
Population characteristics	No work involving human participants or their data or biological material.
Recruitment	No work involving human participants or their data or biological material.
Ethics oversight	No work involving human participants or their data or biological material.

Note that full information on the approval of the study protocol must also be provided in the manuscript.

Field-specific reporting

Please select the one below that is the best fit for your research. If you are not sure, read the appropriate sections before making your selection.

☒ Life sciences ☐ Behavioural & social sciences ☐ Ecological, evolutionary & environmental sciences

For a reference copy of the document with all sections, see nature.com/documents/nr-reporting-summary-flat.pdf

Life sciences study design

All studies must disclose on these points even when the disclosure is negative.

Sample size	For non-mouse experiments, no statistical method was used to predetermine sample size, however we used a minimum of 3 independent biological replicates in accordance with common practice in the field. Power analysis was used for animal experiments to determine number of animals required per group to detect a 30-40% difference in gene expression, structural and functional measurements (SD=0.2, alpha=0.05, confidence=95%).
Data exclusions	Any cases of data exclusion have been outlined in their respective methods section (typically measurements more than two standard deviations from the group mean were excluded on a per-parameter basis).

Replication	For findings from the single-cell RNA-seq datasets, we performed independent qPCR and FACS analyses to confirm reproducibility of the trends seen at the transcript and protein levels.
Randomization	Samples were allocated randomly where possible (cell experiments).
Blinding	Investigators were blinded for mouse phenotyping experiments and outcome assessment.

Reporting for specific materials, systems and methods

We require information from authors about some types of materials, experimental systems and methods used in many studies. Here, indicate whether each material, system or method listed is relevant to your study. If you are not sure if a list item applies to your research, read the appropriate section before selecting a response.

Materials & experimental systems

n/a	Involved in the study
☐	☒ Antibodies
☐	☒ Eukaryotic cell lines
☒	☐ Palaeontology and archaeology
☐	☒ Animals and other organisms
☒	☐ Clinical data
☒	☐ Dual use research of concern
☒	☐ Plants

Methods

n/a	Involved in the study
☒	☐ ChIP-seq
☐	☒ Flow cytometry
☒	☐ MRI-based neuroimaging

Antibodies

Antibodies used	BV421 Rat Anti-SSEA-3 BD Biosciences Cat. #562706<br>TotalSeq-A0251 anti-human Hashtag 1 BioLegend Cat. #394601<br>TotalSeq-A0252 anti-human Hashtag 2 BioLegend Cat. #394603<br>TotalSeq-A0253 anti-human Hashtag 3 BioLegend Cat. #394605<br>TotalSeq-A0254 anti-human Hashtag 4 BioLegend Cat. #394607<br>TotalSeq-A0255 anti-human Hashtag 5 BioLegend Cat. #394609<br>TotalSeq-A0256 anti-human Hashtag 6 BioLegend Cat. #394611<br>TotalSeq-A0257 anti-human Hashtag 7 BioLegend Cat. #394613<br>TotalSeq-A0258 anti-human Hashtag 8 BioLegend Cat. #394615<br>Human VEGFR2/KDR/Flk-1 PE-conjugated Antibody (R&D Systems, #FAB357P)<br>PerCP Mouse Anti-Human CD34 (BD Biosciences, #340430)
Validation	SSEA-3 antibody validated for application on BD Biosciences website in flow cytometry in human cells. TotalSeq hashtag antibodies validated for proteogenomics applications for human cells, each lot is quality tested with flow cytometry and sequencing for the oligomer (stated on BioLegend website). KDR antibody validated for application in flow cytometry (R&D systems website). We have previously validated the CD34 antibody for flow cytometry (PMID:27906170).

Eukaryotic cell lines

Policy information about cell lines and Sex and Gender in Research

Cell line source(s)	WTC WT-11 hiPSCs (Gladstone Institute of Cardiovascular Research, UCSF; Karyotype: 46, XY; RRID: CVCL_Y803, generated as previously described - PMID:24509632) were used to generate barcoding lines.<br>The cell line used to generate the time course and TMEM88 scRNA-seq datasets were WTC CRISPRi TMEM88-g2.3 GCaMP hiPSCs (Karyotype: 46, XY; RRID: CVCL_VM38; generously provided by M. Mandegar and B. Conklin, Gladstone Institute, UCSF), generated as previously described (PMID:26971820).
Authentication	Barcoding lines were each karyotyped after genome editing and selection. TMEM88 CRISPRi line was not authenticated after CRISPRi editing.
Mycoplasma contamination	All barcoding lines were tested for myoplasma and confirmed negative. The TMEM88 CRISPRi line was not tested for myoplasma contamination.
Commonly misidentified lines<br>(See ICLAC register)	No commonly misidentified lines used in the study.

Animals and other research organisms

Policy information about studies involving animals; ARRIVE guidelines recommended for reporting animal research, and Sex and Gender in Research

Laboratory animals	Wildtype and TMEM88 KO C57BL/6 mice aged between 8 and 28 weeks (adult) and E15.5 (embryos) were used in this study.
Wild animals	No wild animals were used in this study.
Reporting on sex	For all experiments, sexes wee pooled in the study design as after normalising for body weight, there did not appear to be any sex-specific differences in the measured parameters. This ideally also improves robustness and generalisability of the results and maximises animal usage for ethics considerations. Sex for each measurement reported is provided in the source data.
Field-collected samples	The study did not involve field-collected samples.
Ethics oversight	All human pluripotent stem cell studies were carried out in accordance with consent from the University of Queensland's Institutional Human Research Ethics approval (HREC#: 2015001434). All mouse breeding was conducted in accordance with consent from the University of Queensland's Molecular Biosciences Animal Ethics committee approval (Molecular Biosciences AEC-MBS approval #: 2018/AE000177 & 2021/AE000421). All mouse experiments were carried out in accordance with consent from the University of Queensland's Molecular Biosciences Animal Ethics committee approval (Molecular Biosciences AEC-MBS approval #: 2018/AE000171 & 2021/AE000999).

Note that full information on the approval of the study protocol must also be provided in the manuscript.

Plants

Seed stocks	No plants used in the study.
Novel plant genotypes	No plants used in the study.
Authentication	No plants used in the study.

Flow Cytometry

Plots

Confirm that:

- [x] The axis labels state the marker and fluorochrome used (e.g. CD4-FITC).
- [x] The axis scales are clearly visible. Include numbers along axes only for bottom left plot of group (a 'group' is an analysis of identical markers).
- [x] All plots are contour plots with outliers or pseudocolor plots.
- [x] A numerical value for number of cells or percentage (with statistics) is provided.

Methodology

Sample preparation	Cells were dissociated with 0.5mM % EDTA + 0.25% Trypsin (1:10) then neutralised with foetal bovine serum (FBS) in DMEM/F12 or RPMI media (1:1). Cells were fixed using 4% paraformaldehyde, permeabilised in 0.75% saponin with 5% FBS in PBS and stained for flow cytometry using cardiac troponin T (Creative Biolabs, #M40283, 1:200 dilution). For live cell cytometry, cells were stained in DMEM/F12 or RPMI media for SSEA3 (BD Biosciences, #562706, 1:20 dilution), KDR (R&D Systems, #FAB357P, 1:10 dilution), or CD34 (BD Biosciences, #340430, 1:5 dilution) with the corresponding immunoglobin G isotype control.
Instrument	BD FACSCANTOII flow cytometer.
Software	FACSDiva software (v9.0) was used to collect the data. FlowJo (v10.07) was used to visualise and analyse the FACS data. Summarised percentages from FACS analysis were analysed and used for statistical testing in R (v3.6.0) with ggpubr (v0.2.3).
Cell population abundance	For all flow cytometry, at least 7000 single cells were collected. Gating strategy and details on post-sort populations are provided in their respective figures.
Gating strategy	We have provided an example of the gating strategy including FSC/SCC to identify starting single cell population in

Gating strategy

Supplementary Figure 6. Gates for defining positive and negative populations are defined in their respective figures, where the gates are defined using the immunoglobin G (IgG) isotype control.

☒ Tick this box to confirm that a figure exemplifying the gating strategy is provided in the Supplementary Information.
