# Supplementary material for: Atlas of multilineage stem cell differentiation reveals TMEM88 as a developmental regulator of blood pressure
Source: Nat Commun. 2025 Feb 4;16:1356. doi: 10.1038/s41467-025-56533-2 (PMC11794859; doi:10.1038/s41467-025-56533-2)
Supplement: Supplementary file 6 — Supplementary Data 3 [file heatmap.pdf › cpdb_results/CHIR_day9_method2/heatmap.pdf]

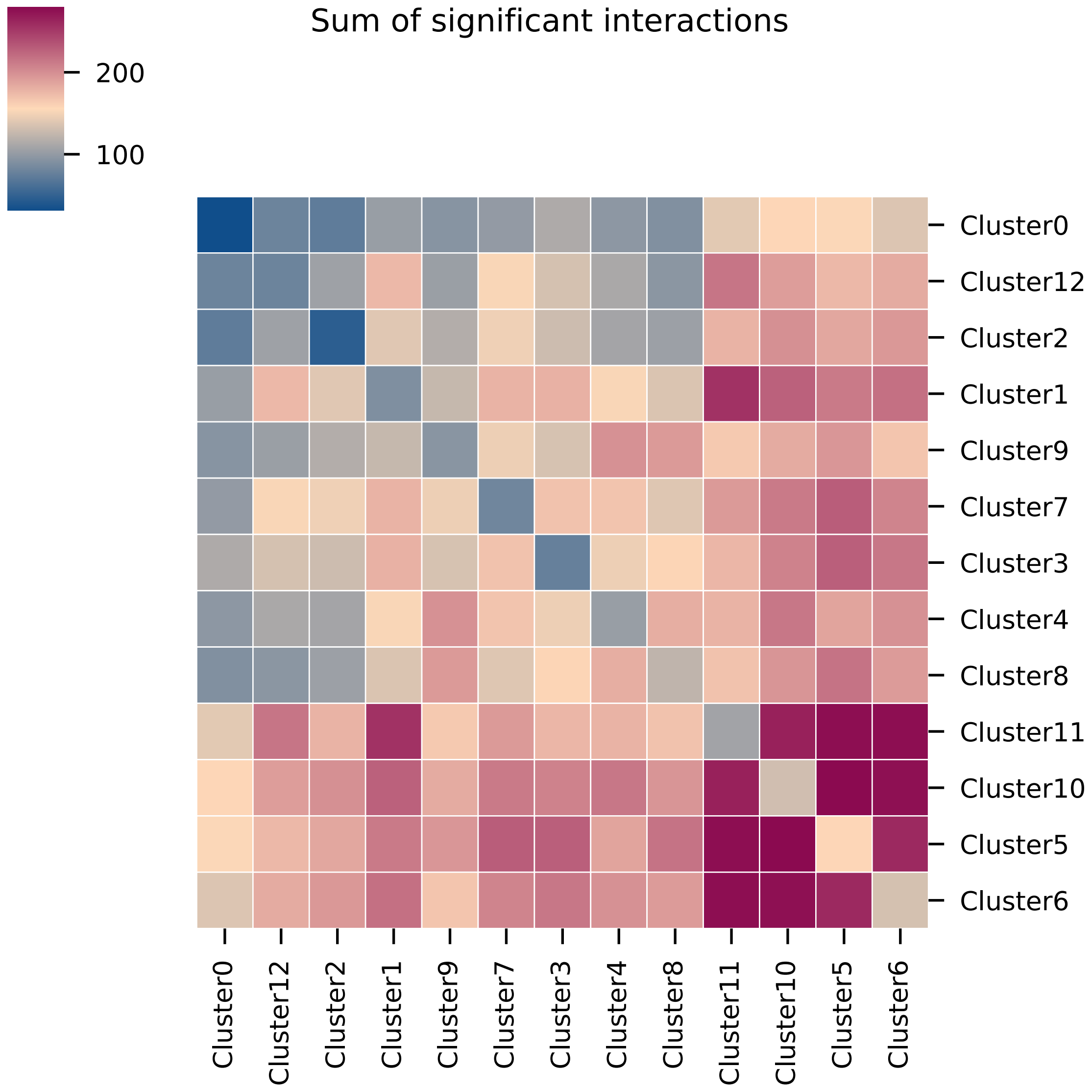

Sum of significant interactions
200
100
Cluster0
Cluster12
Cluster2
Cluster1
Cluster9
Cluster7
Cluster3
Cluster4
Cluster8
Cluster11
Cluster10
Cluster5
Cluster6
