# Supplementary material for: Atlas of multilineage stem cell differentiation reveals TMEM88 as a developmental regulator of blood pressure
Source: Nat Commun. 2025 Feb 4;16:1356. doi: 10.1038/s41467-025-56533-2 (PMC11794859; doi:10.1038/s41467-025-56533-2)
Supplement: Supplementary file 6 — Supplementary Data 3 [file 41467_2025_56533_MOESM6_ESM.zip › cpdb_results/lowDorso_day5_method2/heatmap.pdf]

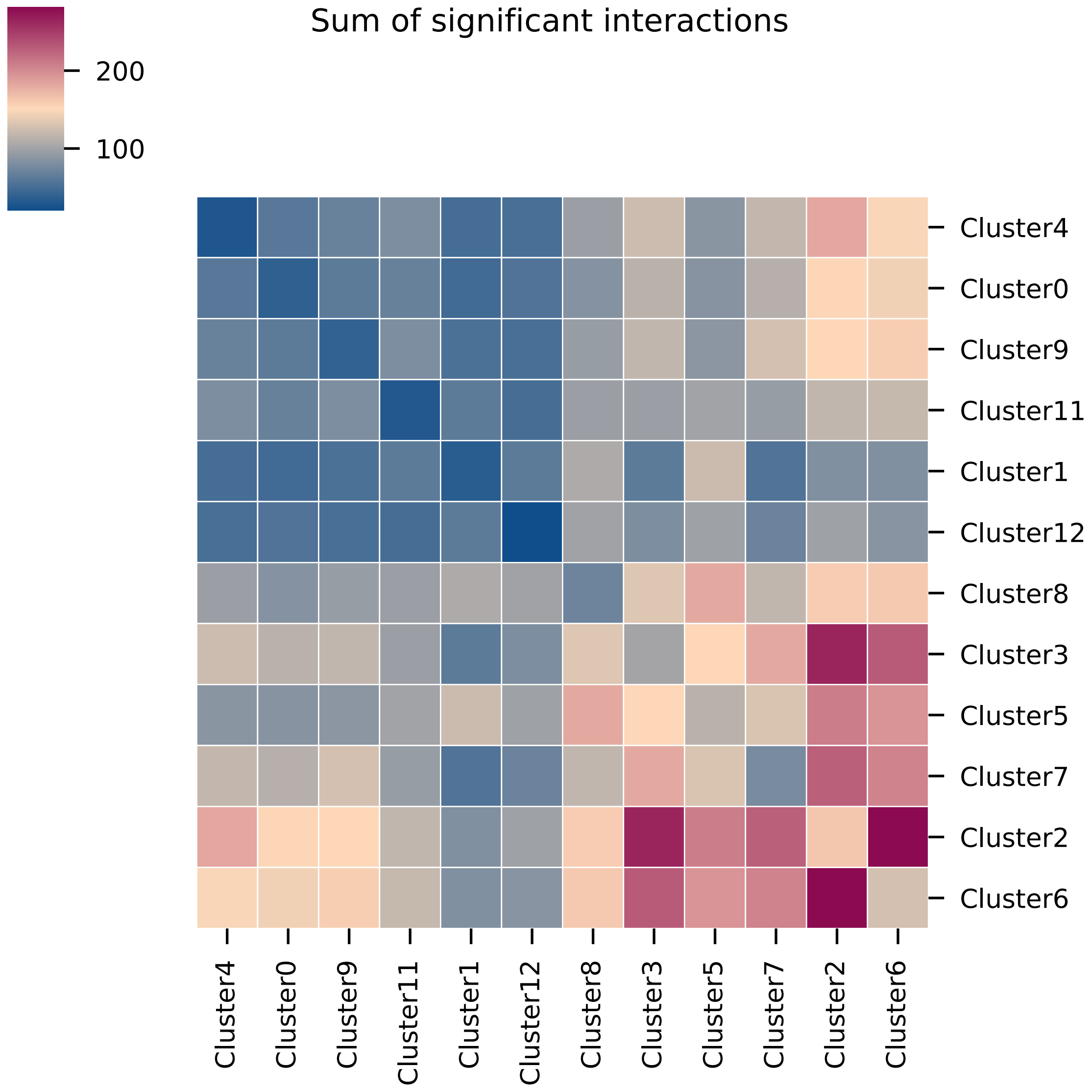
Sum of significant interactions
200
100
Cluster4
Cluster0
Cluster9
Cluster11
Cluster1
Cluster12
Cluster8
Cluster3
Cluster5
Cluster7
Cluster2
Cluster6
