# Supplementary material for: Atlas of multilineage stem cell differentiation reveals TMEM88 as a developmental regulator of blood pressure
Source: Nat Commun. 2025 Feb 4;16:1356. doi: 10.1038/s41467-025-56533-2 (PMC11794859; doi:10.1038/s41467-025-56533-2)
Supplement: Supplementary file 6 — Supplementary Data 3 [file 41467_2025_56533_MOESM6_ESM.zip › cpdb_results/lowDorso_day9_method2/heatmap.pdf]

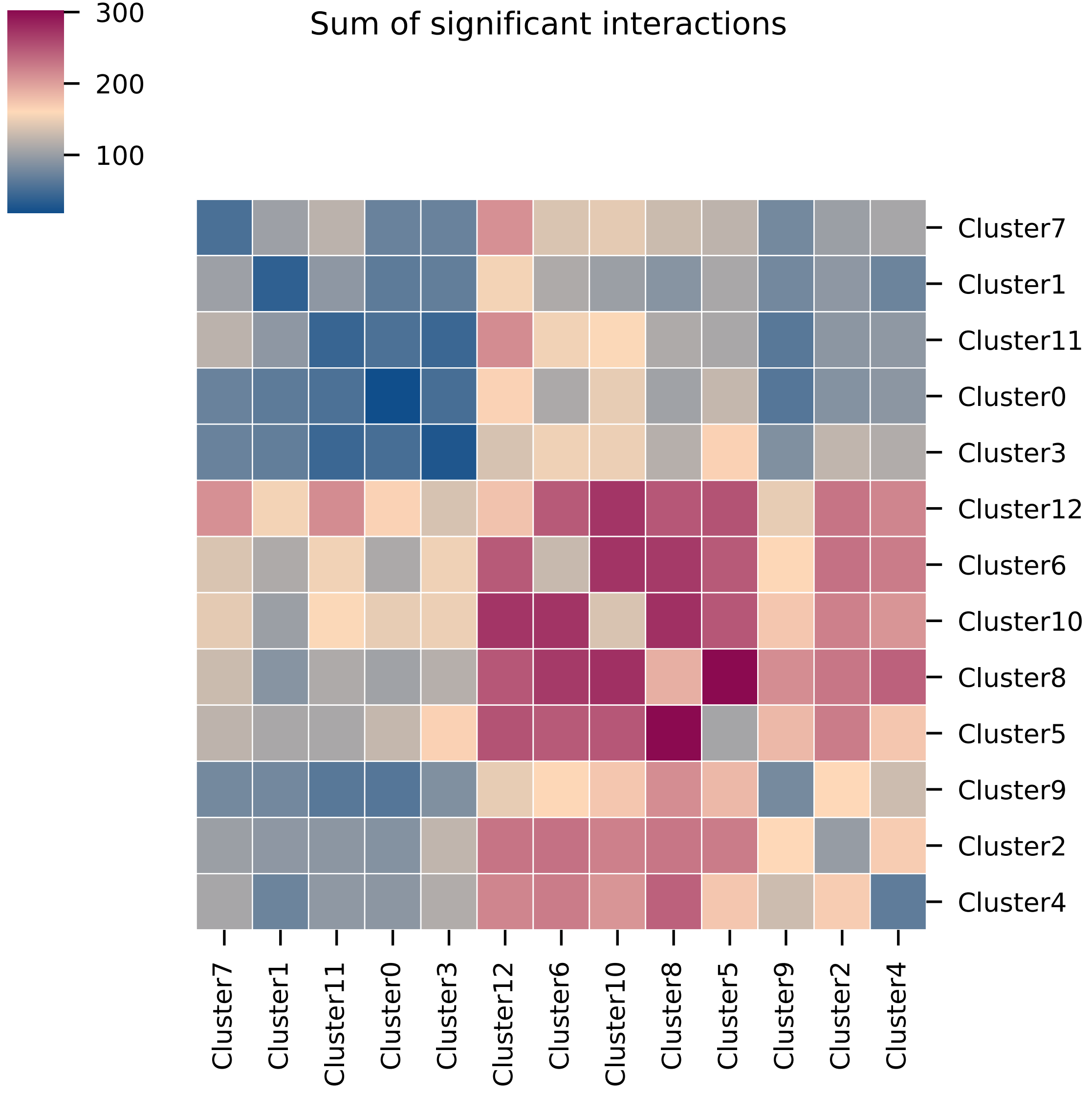
Sum of significant interactions
300
200
100
Cluster7
Cluster1
Cluster11
Cluster0
Cluster3
Cluster12
Cluster6
Cluster10
Cluster8
Cluster5
Cluster9
Cluster2
Cluster4
